# MicroRNAs affecting the susceptibility of melanoma cells to CD8^+^ T cell‐mediated cytolysis

**DOI:** 10.1002/ctm2.1186

**Published:** 2023-01-30

**Authors:** Antonino A. Pane, Theresa Kordaß, Agnes Hotz‐Wagenblatt, Elke Dickes, Annette Kopp‐Schneider, Rainer Will, Barbara Seliger, Wolfram Osen, Stefan B. Eichmüller

**Affiliations:** ^1^ Research Group GMP & T Cell Therapy German Cancer Research Center (DKFZ) Heidelberg Germany; ^2^ Faculty of Biosciences University of Heidelberg Heidelberg Germany; ^3^ Omics IT and Data Management Core Facility German Cancer Research Center (DKFZ) Heidelberg Germany; ^4^ Department of Biostatistics German Cancer Research Center (DKFZ) Heidelberg Germany; ^5^ Core Facility Cellular Tools German Cancer Research Center (DKFZ) Heidelberg Germany; ^6^ Institute of Medical Immunology Martin‐Luther‐University Halle‐Wittenberg Halle/Saale Germany; ^7^ Present address: Immatics Biotechnologies GmbH Tübingen Germany; ^8^ Present address: Section Multiple Myeloma Internal Medicine V, University Clinic Heidelberg Heidelberg Germany

**Keywords:** cytotoxic T lymphocyte (CTL), melanoma, miRNA, Ndufa1, *Psmc3*, screening

## Abstract

**Background:**

The regulatory functions of microRNAs (miRNAs) in anti‐tumour immunity have been mainly described in immune effector cells. Since little is known about miRNA effects on the susceptibility of target cells during T cell—target cell interaction, this study focused on the identification of miRNAs expressed in tumour cells controlling their susceptibility to CD8^+^ T cell‐mediated cytotoxicity.

**Methods:**

Luciferase expressing B16F10 melanoma (B16F10 Luci^+^) cells transfected with individual miRNAs covering a comprehensive murine miRNA library were screened for their susceptibility to lysis by an established cytotoxic T lymphocyte (CTL) line (5a, clone Nβ) specific for the melanoma‐associated antigen tyrosinase‐related protein 2. miRNAs with the most pronounced effects on T cell‐mediated lysis were validated and stably expressed in B16F10 cells. In silico analyses identified common targets of miRNA sets determined by the screen, which were further confirmed by small interfering RNA (siRNA)‐mediated silencing experiments modulating immune surveillance. The Ingenuity Pathway Analysis (IPA) software and RNA sequencing (RNA‐seq) data from miRNA‐overexpressing cell lines were applied to investigate the underlying mechanisms. The Cancer Genome Atlas (TCGA)‐derived miRNA sequencing data were used to assess the correlation of miRNA expression with melanoma patients’ survival.

**Results:**

The miRNA screen resulted in the selection of seven miRNAs enhancing CTL‐mediated melanoma cell killing in vitro. Upon stable overexpression of selected miRNAs, hsa‐miR‐320a‐3p, mmu‐miR‐7037‐5p and mmu‐miR‐666‐3p were determined as most effective in enhancing susceptibility to CTL lysis. In silico analyses and subsequent siRNA‐mediated silencing experiments identified *Psmc3* and *Ndufa1* as common miRNA targets possibly involved in the functional effects observed. The analyses of RNA‐seq data with IPA showed pathways, networks, biological functions and key molecules potentially involved in the miRNA‐mediated functional effects. Finally, based on TCGA data analysis, a positive correlation of the conserved miRNAs among the panel of the seven identified miRNAs with overall survival of melanoma patients was determined.

**Conclusions:**

For the first time, this study uncovered miRNA species that affect the susceptibility of melanoma cells to T cell‐mediated killing. These miRNAs might represent attractive candidates for novel therapy approaches against melanoma and other tumour entities.

## Graphical abstract

– miRNAs that significantly increase susceptibility of melanoma cells to CTL lysis were discovered.
– RNA‐seq analyses show networks and pathways potentially involved in enhanced susceptibility to CTLs.
– TCGA data analyses reveled correlations between expression of certain newly identified miRNAs and patient survival.
– These miRNAs might promote the development of novel cancer therapies.

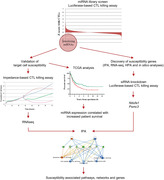

## 1 BACKGROUND

Melanoma is the most aggressive type of skin cancer, and its incidence has been increasing for decades in Caucasian populations.^1^, ^2^ The prognosis of melanoma strongly depends on the stage of the disease at diagnosis. While patients with primary localised tumours can be successfully treated by surgery alone with 5‐year survival rates of more than 90%, the prognosis for patients with metastatic disease is very poor.^1^, ^2^ In the last decade, the US Food and Drug Administration approved small inhibitory molecules and immune checkpoint inhibitors for the treatment of metastatic melanoma.^3^, ^4^ These changes in the treatment landscape have significantly improved patient outcomes, and in particular, immune checkpoint blockade treatments have yielded unprecedented results.^5^, ^6^, ^7^ Relying on the fact that T cells play a prominent role in anti‐tumour immune responses,^8^ this strategy uses monoclonal antibodies directed against T cell inhibitory receptors, most prominently cytotoxic T‐lymphocyte‐associated protein 4 (CTLA‐4) and programmed cell death protein 1 (PD‐1) and its ligand PD‐L1, to enhance T cell‐mediated anti‐tumour effector functions.^9^ Currently, anti‐PD‐1 blockade as monotherapy or combined with anti‐CTLA‐4 antibodies is recommended as first‐line treatment for stage IV unresectable melanoma.^3^, ^4^ Nevertheless, these treatments present limitations related to their adverse side effects and limited efficacies^7^, ^10^ caused by escape mechanisms, including loss or downregulation of major histocompatibility complex (MHC) class I molecules or antigen processing machinery (APM) components and molecules involved in interferon (IFN)γ signalling,^11^, ^12^ leading to evasion from cytotoxic T lymphocyte (CTL) lysis.^8^, ^13^ Currently, new approaches are under investigation to enhance the clinical efficacy of cancer immunotherapies, such as the use of microRNAs (miRNAs) as regulators of expression of immune regulatory genes.

miRNAs are single‐stranded small RNAs of 19–24 nucleotides in length mainly binding to the 3′ untranslated region of their mRNA targets,^14^, ^15^ ultimately leading to gene silencing through different mechanisms.^16^ In general, the effect of a miRNA molecule on protein expression is at the twofold level, and due to its promiscuous properties, a given miRNA can modulate the expression of hundreds of proteins, while a single transcript can be targeted by many miRNAs.^17^, ^18^ Apart from the key role miRNAs play in physiologic processes, the miRNA expression pattern can be altered by pathophysiologic conditions, including cancer. miRNAs can act as tumour‐promoting miRNAs (oncomiRs) or as tumour suppressors in different tumour types. For example, our group has shown that miR‐576‐5p enhances invasion of human melanoma cell lines in vitro, whereas miR‐193b and miR‐30c‐1* cause the opposite effect.^19^ Furthermore, in human melanoma, miR‐339‐3p was reported to act as a tumour suppressor reducing invasion,^20^ while miR‐137 was shown to inhibit cell migration, invasion and proliferation, and also to induce apoptosis.^21^ Interestingly, miRNAs also participate in the regulation of immune processes and could therefore be involved in tumour immune surveillance and escape.^22^ These include miRNAs targeting the non‐classical human leukocyte antigen (HLA)‐G, PD‐L1, PD‐L2 and HLA class I APM components,^23^, ^24^, ^25^, ^26^, ^27^ which were associated with altered immune responses and clinical parameters. Moreover, miRNAs targeting NT5E, a nucleotidase expressed on the cell surface that hydrolyses extracellular adenosine monophosphate to generate adenosine, thereby inhibiting cellular immune responses, have been described. The miRNAs targeting NT5E or its transcription factors in cancer might influence the tumour microenvironment and affect the anti‐tumoural immune responses.^28^ Despite the fact that a number of miRNAs, such as miR‐222, miR‐339 and miR‐210, reduce the susceptibility of tumour cells to CTL‐mediated cytotoxicity,^29^, ^30^ little is known about how miRNAs expressed in tumour cells affect their susceptibility to T cell‐mediated tumour cell killing.

Based on these results, it is postulated that miRNAs might represent potential biomarkers for diagnosis and prognosis as well as therapeutic targets or tools. Recently, miRNA‐based treatments have reached clinical trials.^31^ For example, tumour suppressor miR‐34 mimics were administered as liposome formulations to treat different tumours,^32^ and miR‐155 was inhibited with the MRG‐106 molecule for the treatment of cutaneous T cell lymphoma.^31^ Hence, our aim of identifying miRNAs involved in tumour cell—T cell interactions may be of crucial importance for the development of new miRNA‐based therapeutic approaches against cancer. In this study, we performed a comprehensive high‐throughput functional screen that identified novel miRNA species affecting the susceptibility of melanoma cells to CTL‐mediated killing. Moreover, RNA sequencing (RNA‐seq) and in silico pathway analyses identified pathways, networks and key molecules whose miRNA‐mediated dysregulation might explain the functional effects observed.

## 2 METHODS

### 2.1 Luciferase‐based cytotoxicity assay

The luciferase‐based cytotoxicity assay was performed as described by Khandelwal et al.^33^ with modifications. In its 4‐day version, tyrosinase‐related protein 2 (TRP‐2)‐specific CTLs (line 5a, clone Nβ; or fresh medium) were co‐cultured with 2000 target cells that had been transfected (Metafectene SI^+^ reagent, Biontex) with mi/small interfering RNA (siRNA) 3 days before (50–100 nM, as indicated for each case). After 21 h, the wells were rinsed with phosphate‐buffered saline followed by addition of 20 μl/well of lysis buffer. After a 15‐min incubation at room temperature, 100 μl/well of luciferase buffer was added, and the luminescence was measured using an LB940 Multimode Reader Mithras (Berthold Technologies) (Figure S1A). The means of the replicates’ relative luminescence unit (RLU) values show the miRNA effects on the viability per se (without CTLs) or on the susceptibility to CTL killing (with CTLs). The fractional killing [FK = (RLU_without CTLs_ – RLU_with CTLs_)/RLU_without CTLs_)] was calculated with Excel. Using an R code created for this study, *t*‐tests were used to compare the FK values with a control (indicated in each case). Pre‐tests were performed before the assays (same week) to determine the CTL numbers/well required to achieve 50% of maximum cytolysis, which were then used in each experiment. This assay was also adapted to test the B16F10 Luci^+^ chicken ovalbumin (OVA^+^) cells stably transduced with miRNAs (2‐day version) in comparison to a control cell line (B16F10 miR‐Neg. Control, clone L), where 8000 tumour cells/well were seeded and 36 000 CTLs/well were added after 24 h. For the validation assays, four replicates of each condition per plate were used. If wells were discarded due to contamination or technical error, three wells were used for calculation.

### 2.2 miRNA high‐throughput screen

Using the luciferase‐based killing assay in the 4‐day version (see above) (Figure S1A), the mirVana miRNA Mimic Library, Pre‐Defined Mouse version 20 (Thermo Fischer Scientific) was screened. Four plates were assayed from each miRNA library 96‐well plate, two of them receiving CTLs and the other two only receiving medium; hence, each miRNA is associated with four RLU values (each representing one well in a different plate). Four miRNAs that repeatedly led to specific effects on the cell viability per se and CTL‐mediated cytolysis in previous experiments were used as controls for the screen (see extra methods in Supporting Information) to test for reproducibility and robustness in the screen quality control. A concentration of 50 nM miRNA and 48 000 CTLs/well were used unless otherwise indicated. The data were analysed with the CellHTS2 R package (Bioconductor).^34^ The code normalises the raw data (RLU) to the median of each particular plate (intra‐plate normalisation), thereby enabling comparison of all plates from the screen. This normalisation considers that most of the miRNAs have no significant effect on killing or viability (which can be confirmed once the data are available). After this step, it is impossible to compare results from plates receiving CTLs with the ones receiving only medium (as the medians used in normalisation are theoretically different). Afterwards, RLU *Z*‑scores with and without CTLs were calculated for each miRNA [*Z*‑score*_x_* = (normalised RLU*_x_* – median normalised RLU)/mean absolute deviation]. Quality control of all plates in the screen was performed by using the *Z*‐scores of the controls included in each plate. Position effects were corrected with the LOESS regression and the mathematical sign of all the values was inverted in the code (positive values refer to an increase in the killing and negative ones to a decrease). miRNAs resulting in *Z*‐scores without CTLs ≥|1| were discarded, whereas miRNAs showing strong effects on cytolysis (*Z*‐scores with CTLs ≥|1|) were preselected (53 species) and subjected to two additional validation luciferase experiments. RLU *Z*‐scores, FK values and FK *Z*‐scores were calculated and used in a multistep ranking process: (i) the miRNAs showing the opposite effect compared to the screen results were discarded; (ii) the miRNAs with FK *Z*‐scores <|0.9| were discarded; (iii) the *ranked products* ^35^ analysis was used to further rank the preselected miRNAs; and (iv) priority was given to the miRNAs confirmed in both validation assays using both types of analysis (RLU *Z*‐scores and FK *Z*‐scores). The remaining 16 miRNAs were tested three more times with the luciferase‐based cytotoxicity assay, and these results were used to narrow down the list to the top seven candidates.

### 2.3 In silico establishment of the predicted screen‐wide enriched miRNA target genes list

Based on the results of the miRNA library screen, the miRNAs were categorised into two groups: miRNAs with increased CTL‐mediated cytolysis (IK, the 50 miRNAs that showed the highest positive *Z*‐scores with CTLs) and those with no effect on target cell lysis (NE, the 100 miRNAs showing the lowest |*Z*‐scores| in the screen, all of which had |*Z*‐scores| ≤0.04). Different mouse miRNA target prediction databases (TargetScan,^36^, ^37^ microRNA consv. and non‐consv.^38^, ^39^ PITA,^40^, ^41^ PITA_all^40^, ^41^ and MicroCosm^42^) were employed to determine the times each gene was regulated by each miRNA group. Using Fisher's exact tests for each gene (obtaining odds ratios and *p*‐values) allowed the selection of the targets that were enriched in the IK target gene group (i.e., highly targeted by the IK miRNAs and not targeted by the NE miRNAs), leading to an IK enriched target gene list. These genes were ranked according to *p*‐values, and the genes targeted by at least five miRNAs from the IK group were preselected. This analysis could not be performed with the miRNAs that decreased the killing due to the low number of candidates showing significant effects. These preselected miRNA targets were further subjected to IPA *Core Analyses*, expression analyses using RNA‐seq datasets in B16F10 (SRA, NCBI, Table S12) and analyses using the Human Protein Atlas (HPA) (Figure S2), which are further described in Section 3.

### 2.4 IPA software analyses of RNA‐seq data

RNA‐seq data were analysed using IPA (QIAGEN Inc., https://www.qiagenbioinformatics.com/products/ingenuitypathway‐analysis).^43^ For the IPA individual *Core Analyses*, the RNA‐seq data from each cell line's comparison with control was uploaded to IPA using a cut‐off of *p*‐value = .001 and fold change (FC) = 2/0.5(log_2_(FC) = |1|). The data were analysed using IPA *Core Analyses* including all predicted miRNA targets (IPA database) and the default settings. The *Canonical Pathways* were filtered with a cut‐off of *p*‐value = .05 and included the high‐order categories: *Apoptosis*, *Cancer*, *Cellular Immune Response*, *Cellular Growth*, *Proliferation and Development* and *Cellular Stress and Injury*. Irrelevant pathways were discarded (e.g., non‐murine/human pathways), whereas those with the lowest *p*‐values and highest |*Z*‐scores| were further analysed. The *Canonical Pathway Antigen Presentation* was analysed for every cell line. The same filtering strategy as with *Canonical Pathways* was used for the *Diseases and Biological Functions* analysis, although in this case, the filtering included the categories: *Diseases and Disorders* and *Molecular and Cellular Functions*, and further fine filtering was done to include all the low‐order categories that were relevant. High‐order categories that were significantly regulated (e.g., *Cancer*) were selected and in‐depth analysis of the lower order functions was performed. For the *Regulator Effects* IPA function, thresholds of *p*‐value = .05 and *Z*‐score = |2| were used, and the same pathway filters and processing as with the *Diseases and Biological Functions* analyses were applied. Pathways or functions with the highest scores, lowest *p*‐values and highest relevance were selected, and the IPA *Molecule Activity Predictor* function was used to determine their predicted activation states based on the RNA‐seq expression data, the miRNA target predictions and the experimentally observed data from the Qiagen Knowledge Base. When analysing networks, the IPA *Grow tool* was used to add diseases and functions that were significantly regulated by the molecules in the network. The IPA *Comparison Analysis* was performed comparing the individual *Core Analyses*. The *Molecules, Canonical Pathways* and *Diseases and Biological Functions* analyses were used in search of common annotations affected by all the miRNAs analysed. Pathways or diseases that were regulated by only one miRNA or that were irrelevant were discarded. The same cut‐offs and filtering strategies as with the individual *Core Analyses* were used, with the difference that in the *Diseases and Biological Functions*, a cut‐off of *Z*‐score = |2| was also included. The compared annotations were ordered in decreasing total |*Z*‐score| values and heatmaps were generated.

Licenses were acquired for the software that required it.

*Note*: The human mature miRNAs described are conserved in mice.

Further methods are described in Supporting Information.

## 3 RESULTS

### 3.1 A high‐throughput screen reveals miRNAs affecting the CTL‐mediated lysis of B16F10 cells

**FIGURE 1:**
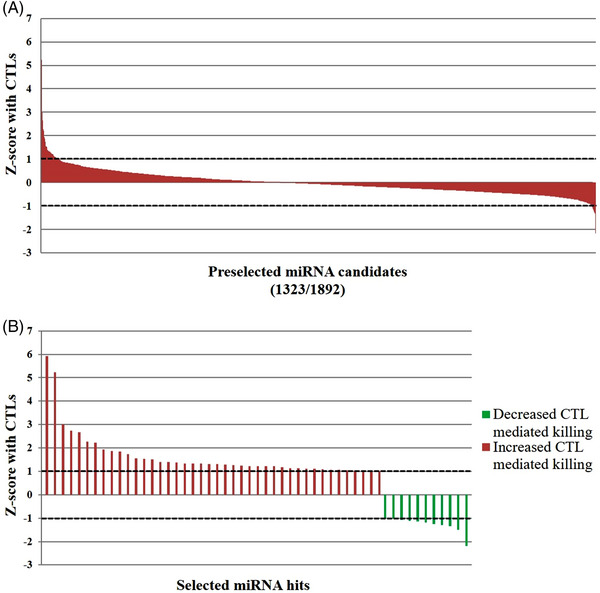
MicroRNA (miRNA) library screen on B16F10 cells with a luciferase‐based cytotoxicity assay. (A) Luciferase assay screen: 2000 B16F10 luciferase^+^ (Luci^+^) cells/well were separately transfected with each individual miRNA (50 nM) for 3 days and 48 000 tyrosinase‐related protein 2 (TRP‐2)‐specific cytotoxic T lymphocytes (CTLs) were added to the corresponding wells. Using the 1323 miRNA candidates from the screen without effects on the viability per se, a threshold of cytotoxicity *Z*‐score with CTLs ≥ |1| (dashed lines) was applied to narrow down the candidates for further validation. (B) miRNAs with *Z*‐scores above threshold showing increased (red) or decreased (green) killing are depicted and furthermore listed in Table S1.

**FIGURE 2:**
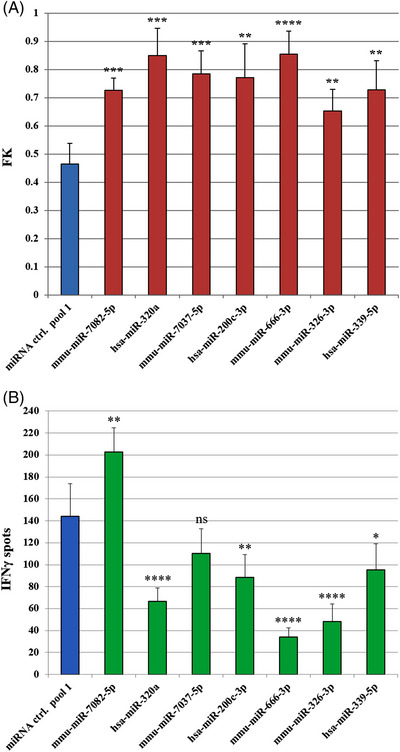
Validation with luciferase‐based cytotoxicity assay of selected microRNAs (miRNAs) and interferon (IFN)γ enzyme‐linked immunosorbent spot (ELISpot) assay. (A) Luciferase assay: 2000 B16F10 luciferase^+^ (Luci^+^) cells/well were transfected with the indicated miRNAs (100 nM) and after 3 days, 96 000 tyrosinase‐related protein 2 (TRP‐2)‐specific cytotoxic T lymphocytes (CTLs) were added to the corresponding wells. Bar graphs show the fractional killing (FK, proportional to the fraction of killed cells) and standard deviation (SD) values of the selected top candidate miRNAs (red) compared to miRNA ctrl. pool 1 (negative control, blue) using two‐sided *t*‐tests (*t*‐test significance with *p*‐values adjusted for multiple testing using the Holm method: ^**^ *p* ≤ .01, ^***^ *p* ≤ .001, ^****^ *p* ≤ .0001). The results shown are representative of three different experiments. (B) IFNγ ELISpot assay: B16F10 Luci^+^ cells transfected for 3 days with the indicated miRNAs (100 nM) were seeded (12 500/well) and co‐cultured with TRP‐2‐specific CTLs (12 500/well) in 96‐well ELISpot plates. Each bar represents the replicates mean of the IFNγ spots and their SDs. Each miRNA was compared to miRNA ctrl. pool 1 using one‐way analysis of variance (ANOVA) (multiplicity adjusted *p*‐values: ^*^ *p* ≤ .05, ^**^ *p* ≤ .01, ^****^ *p* ≤ .0001, ns: not significant). The result from mmu‐miR‐7082‐5p is representative of three different experiments. Human miRNAs shown here are conserved in mice.

In order to identify murine miRNAs that affect the susceptibility of melanoma cells to CTL lysis, B16F10 Luci^+^ cells transiently transfected with individual miRNAs of a comprehensive murine miRNA library were used as target cells for a CTL line specific for the melanoma‐associated tumour antigen TRP‐2. This murine setting was chosen because it enabled screening of the comprehensive miRNA library within a syngeneic setting of melanoma cells and TRP‐2‐specific CTLs, as well as performance of future in vivo studies in C57BL/6 mice. The miRNA effects were analysed in the presence and absence of CTLs using a luciferase‐based cytotoxicity assay (Figure S1B). By excluding miRNAs affecting target cell viability without CTL co‐culture, 1323 miRNAs were preselected for the subsequent analysis. miRNAs were then ranked according to their quantitative effects on CTL‐mediated cytotoxicity determined in luciferase‐based assays (*Z*‐scores with CTLs) (Figure 1A). Only a minority of the miRNAs affected the susceptibility to CTL lysis above the cut‐off (*Z*‐score with CTLs ≥|1|). While 42 of these miRNAs enhanced CTL‐mediated lysis of B16F10 Luci^+^ cells, 11 miRNAs decreased killing of the target cells (Figure 1A,B and Table S1). These miRNAs were tested in confirmatory luciferase‐based cytotoxicity assays and subsequently ranked according to intensity, consistency and statistical significance of their effects on CTL‐mediated lysis. This resulted in the selection of seven top‐ranked miRNAs: mmu‐miR‐7082‐5p, hsa‐miR‐320a‐3p, mmu‐miR‐666‐3p, hsa‐miR‐200c‐3p, mmu‐miR‐326‐3p, mmu‐miR‐7037‐5p and hsa‐miR‐339‐5p (Table S2 and Figure 2A).

**FIGURE 3:**
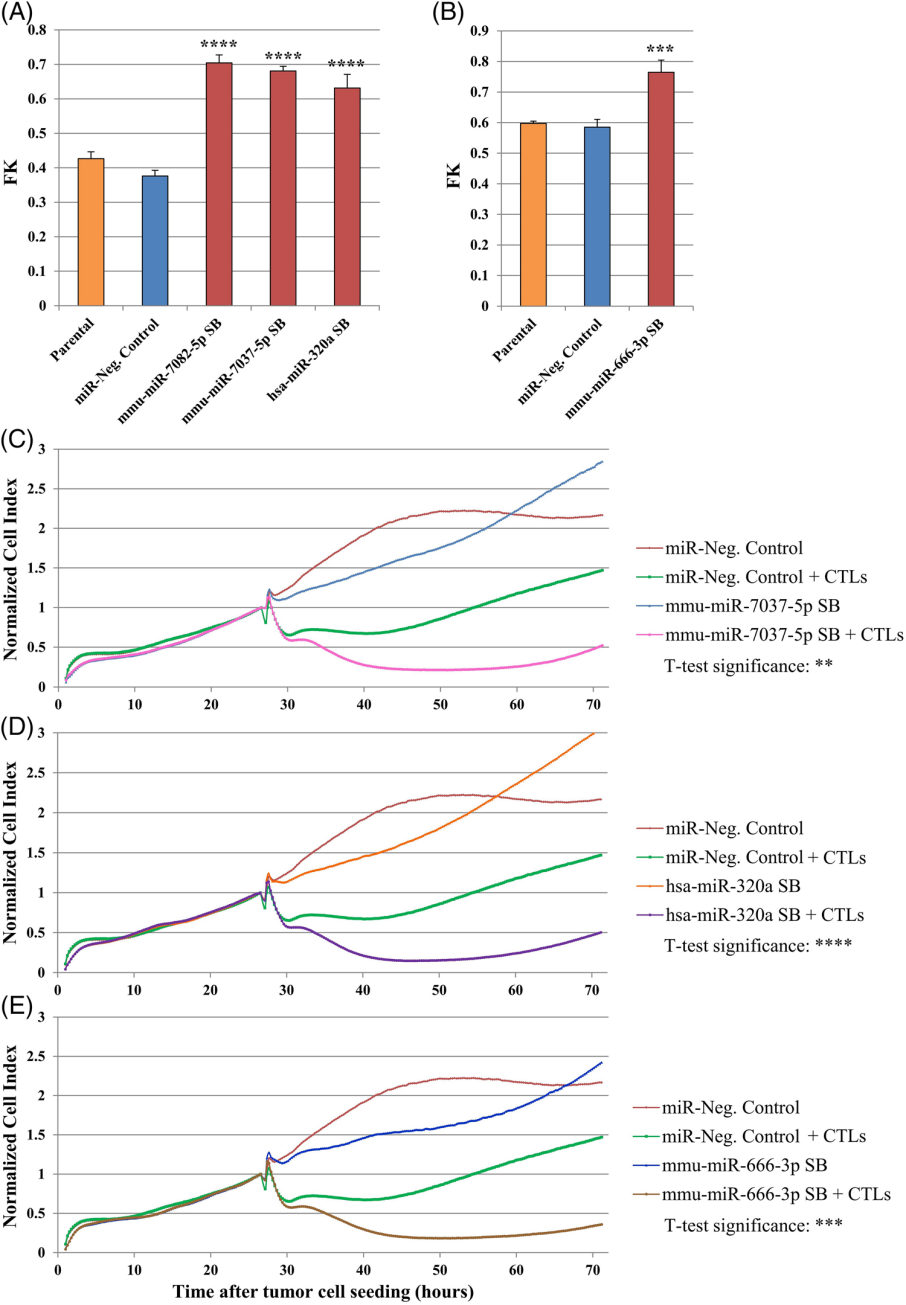
Validation of selected microRNAs (miRNAs) in stably transduced B16F10 sorted bulk (SB) cell lines. (A and B) Luciferase assay, two‐day version: 8000 cells/well of the parental cell line [B16F10 luciferase^+^ (Luci^+^) chicken ovalbumin^+^ (OVA^+^)] or of stable miRNA‐expressing SB cell lines were seeded and 36 000 tyrosinase‐related protein 2 (TRP‐2)‐specific cytotoxic T lymphocytes (CTLs) were added to the corresponding wells. The fractional killing (FK) values from the different cell lines (red) were compared to B16F10 miR‐Neg. Control (blue) using two‐sided *t*‐tests (*t*‐test significance with *p*‐values adjusted for multiple testing using the Holm method: ^***^ *p* ≤ .001, ^****^ *p* ≤ .0001). Panels (A) and (B) represent assays performed in different plates, so they are depicted separately. (C–E) Impedance‐based cytotoxicity assay (xCELLigence): same set up as in (A) and (B). The extent of cytotoxicity observed in the miRNA‐expressing cell lines was compared to B16F10 miR‐Neg. Control with *t*‐tests using an R code (*t*‐test significance with *p*‐values adjusted for multiple testing using the Holm method: ^**^ *p* ≤ .01, ^***^ *p* ≤ .001, ^****^ *p* ≤ .0001). All results are representative of three different experiments. Cell index: proportional to live tumour cell count.

We tested whether the increased killing caused by these miRNAs (Figure 2A) was correlated with enhanced IFNγ secretion by the cognate CTL line in an enzyme‐linked immunosorbent spot (ELISpot) assay (as a surrogate marker for CTL activation). Only mmu‐miR‐7082‐5p led to enhanced IFNγ secretion by the CTLs (Figure 2B), indicating that this could be the cause of the increased killing detected by the luciferase assays with this miRNA. In contrast, transfection of the remaining miRNAs either led to non‐significant changes or to reduced levels of IFNγ secretion, pointing against increased T cell activation as the underlying mechanism of the enhanced target cell cytotoxicity (Figure 2B). In this study, we focused on the effects of miRNAs on susceptibility to T cell‐mediated killing, regardless of the T cell activation status. Hence, enhanced T‐cell IFNγ secretion was not used as a criterion to select the best performing miRNAs for further studies. For further functional validation, the selected miRNAs from Table S2 were lentivirally expressed in B16F10 Luci^+^ cells co‐expressing OVA as optional model antigen for future studies (B16F10 Luci^+^ OVA^+^ cells). In this step, hsa‐miR‐339‐5p was excluded as it showed the lowest ranking in the list. Using fluorescence‐activated cell sorting, clones and sorted bulk (SB) cell lines with stable miRNA overexpression were established, as confirmed by miRNA reverse transcription‐quantitative polymerase chain reaction (RT‐qPCR) analyses (Table S3). In luciferase‐based cytotoxicity assays, mmu‐miR‐7082‐5p, hsa‐miR‐320a‐3p, mmu‐miR‐666‐3p and mmu‐miR‐7037‐5p SB‐expressing cell lines showed consistent and significant increases in susceptibility to CTL‐mediated killing when compared to cell lines expressing a control miRNA (Figure 3A,B), confirming the results observed with the transiently transfected miRNA mimics (Figure 2A). The SB cell lines expressing hsa‐miR‐200c‐3p and mmu‐miR‐326‐3p showed variable results (data not shown) and were thus excluded from further analysis. Finally, the SB cell lines were tested in real‐time impedance‐based CTL cytotoxicity assays and were ranked based on the intensity, statistical significance and consistency of CTL‐mediated cytolysis. The cell lines overexpressing miR‐7037‐5p, miR‐320a‐3p and miR‐666‐3p performed best when compared to control cell lines (Figure 3C–E) and were selected for the subsequent analyses.

### 3.2 Screen‐wide in silico analysis uncovers predicted miRNA targets affecting CTL‐mediated lysis

In parallel, an in silico analysis was performed on the screen results to uncover target molecules that could be involved in the effects observed. We searched for genes that were common targets of the 50 miRNAs leading to the highest increases in CTL‐mediated killing in the screen, yielding a preselection of miRNA targets (see Section 2 and Supporting Information). These targets were further analysed with IPA *Core Analyses*, expression analyses using RNA‐seq datasets in B16F10 cells and analyses using the HPA. The analyses with IPA led to a list of ranked networks, in which these genes were interconnected, allowing for the selection of three networks containing the highest number of predicted miRNA target genes (cut‐off: target genes ≥5; network IDs: 1, 2 and 3 in Table S9). The HPA analysis provided protein expression values in melanoma and other human tumour types, allowing for further ranking of the candidates, with priority given to candidates expressed in melanoma patients. Integrating all the data from these analyses, and discarding candidates not expressed in B16F10 cells, the top‐ranked target genes chosen for further experimental testing were *Ftl1*: ferritin light chain 1, *Ndufa1*: NADH dehydrogenase [ubiquinone] 1 alpha subcomplex subunit 1, *Psmc3*: 26S proteasome regulatory subunit 6A and *Tubb6*: Tubulin beta‐6 chain. Table S10 summarises the results of the RNA‐seq and HPA analyses of these four molecules. Interestingly, these molecules intertwine in the same IPA network (network 2 in Table S9), in which the tumour suppressor protein p53 (TP53) plays a central role (Figure S3).

**FIGURE 4:**
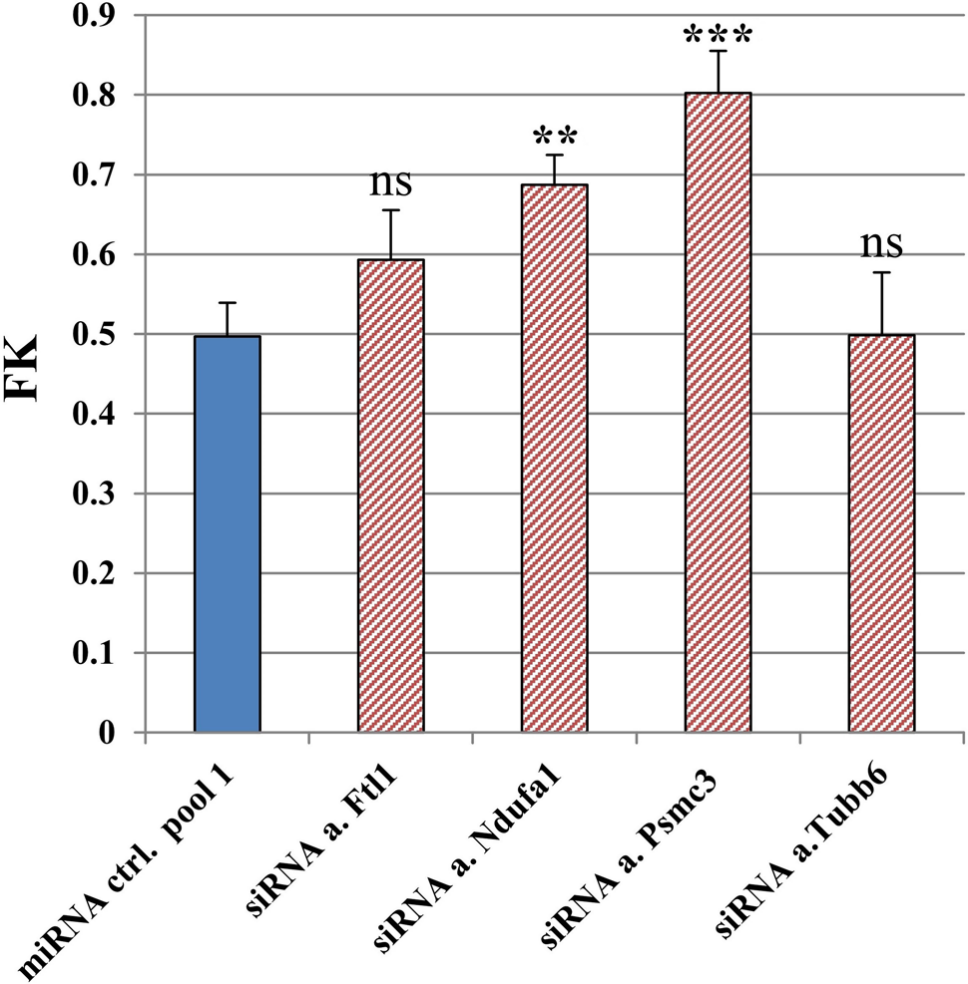
Luciferase assay with small interfering RNA (siRNA) knockdown of selected microRNA (miRNA) targets. A total of 2000 B16F10 luciferase^+^ (Luci^+^) cells/well were transfected with the indicated si/miRNAs (100 nM) for 3 days and 48 000 tyrosinase‐related protein 2 (TRP‐2)‐specific cytotoxic T lymphocytes (CTLs) were added to the corresponding wells. Bar graphs show the fractional killing (FK) and standard deviation (SD) values from the tested siRNA candidates (red stripes) compared to miRNA ctrl. pool 1 (negative control, blue) using two‐sided *t*‐tests (*t*‐test significance with *p*‐values adjusted for multiple testing using the Holm method: ^**^ *p* ≤ .01, ^***^ *p* ≤ .001, ns: not significant). The results shown are representative of three different experiments.

To investigate whether the downregulation of the selected genes affected CTL‐mediated killing as suggested by the in silico analysis, these molecules were targeted with siRNAs. After confirming siRNA‐mediated target gene downregulation with RT‐qPCR (Table S11), the luciferase‐based killing assay was employed to assess the effects of these siRNAs on CTL‐mediated killing. siRNA‐mediated downregulation of *Psmc3* and *Ndufa1* resulted in consistent and significant increased FK, while the remaining two siRNAs led to variable results (Figure 4). In summary, these results provide evidence supporting a role of *Psmc3* and *Ndufa1* in the susceptibility of melanoma cells to CTL‐mediated killing.

### 3.3 RNA‐seq analysis uncovers pathways, functions and networks dysregulated by the selected miRNAs

**FIGURE 5:**
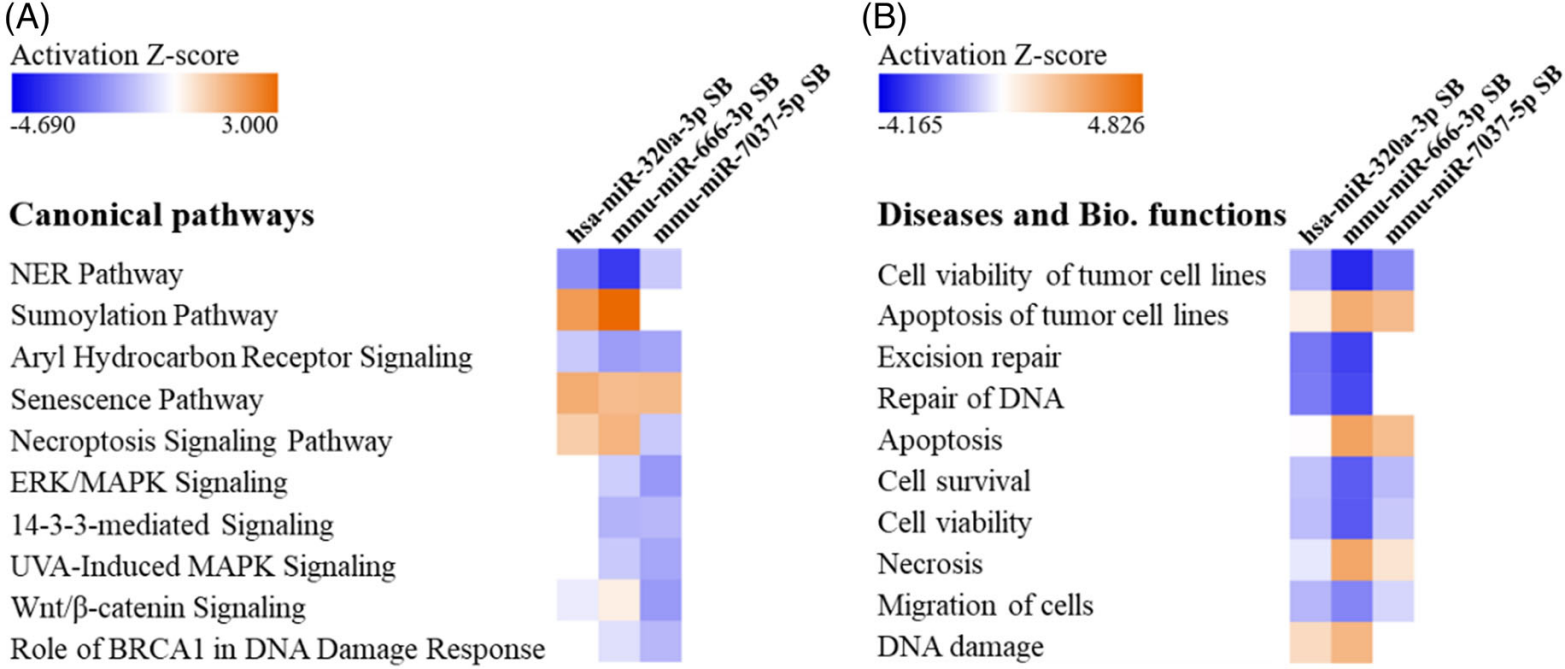
RNA sequencing (RNA‐seq) data Ingenuity Pathway Analysis (IPA) *Comparison Analysis*. The 10 top‐ranked *Canonical Pathways* (A) and *Diseases and Biological Functions* (B) are displayed, indicating the intensity of the microRNA (miRNA)‐specific regulation in each cell line. Figure based on IPA graphs, Qiagen. NER: nucleotide excision repair.

RNA‐seq performed on the selected cell lines B16F10 miR‐7037‐5p^+^ SB, B16F10 miR‐320a‐3p^+^ SB and B16F10 miR‐666‐3p^+^ SB revealed as significantly dysregulated expression of 816, 578 and 1633 genes versus control, respectively (Table S4). The gene expression FC values of these three cell lines versus a control cell line were uploaded to IPA in order to perform different analyses. An IPA *Comparison Analysis* (see Section 2) uncovered commonalities among the individual cell lines. *Nucleotide Excision Repair (NER)*, *Aryl Hydrocarbon Receptor Signalling* (both being inhibited) and the *Senescence Pathway* (which was activated) (Figure 5A), as well as *Diseases and Biological Functions* such as inhibited *Cell Viability* and *Cell Survival* (Figure 5B) were predicted to be consistently dysregulated in all three tested cell lines. However, in some cases the *Z*‐scores associated with each pathway or function varied significantly among the cell lines. For example, *NER* was predicted to be much less downregulated in B16F10 miR‐7037‐5p^+^ SB than in the other two cell lines (Figure 5A), showing that it is less relevant in this case. The dysregulation of these pathways and functions appears in line with the increased susceptibility to CTL lysis observed in the functional experiments.

#### 3.3.1 Dysregulated relevant molecules, antigen presentation and proteasome function

Due to the central role of antigen processing and MHC class I‐restricted epitope presentation in CTL recognition of target cells, the regulation of the *Antigen Presentation Pathway* and the expression of the target antigen TRP‐2, were assessed in each cell line using IPA individual *Core Analyses* (see Section 2). As siRNA knockdown of the predicted miRNA targets *Ndufa1* and *Psmc3* (which is a proteasome subunit) led to an increase in CTL cytotoxicity (Figure 4), these and other associated molecules were also assessed.

**FIGURE 6:**
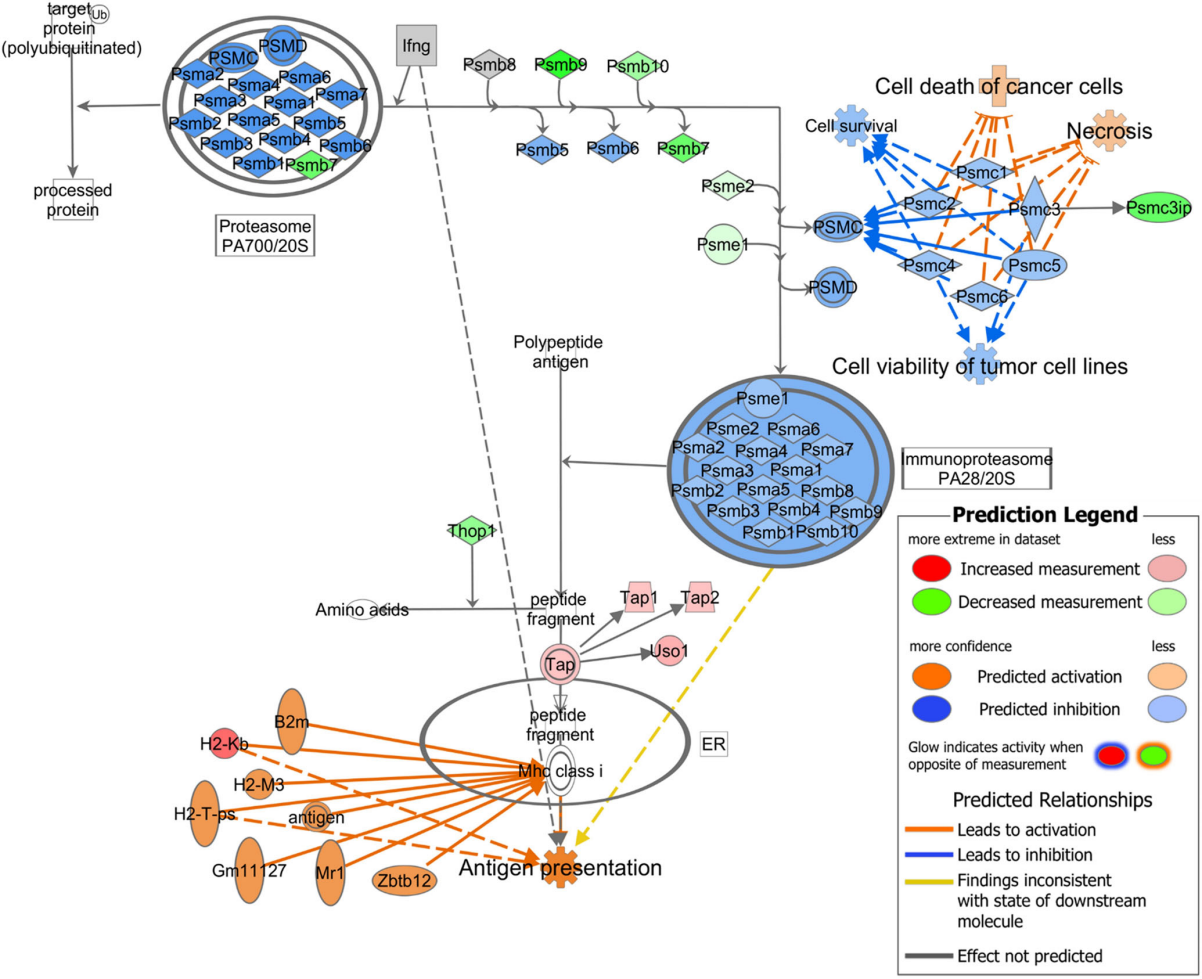
Proteasome and antigen presentation in B16F10 miR‐666‐3p^+^ sorted bulk (SB). The proteasome components and their connection with antigen presentation are depicted. Figure based on Ingenuity Pathway Analysis (IPA) graphs, Qiagen.

**FIGURE 7:**
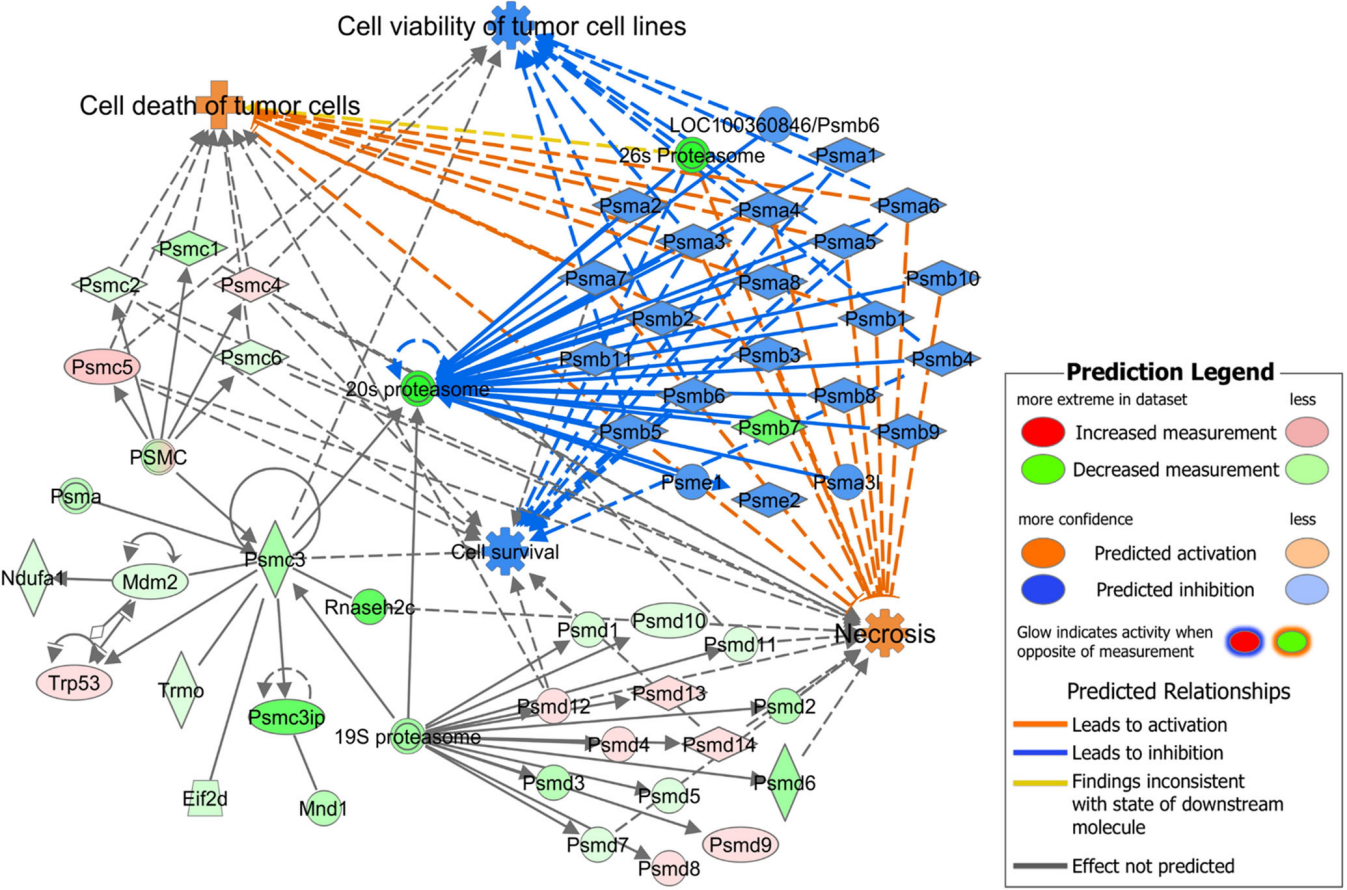
*Psmc3* network in B16F10 miR‐666‐3p^+^ sorted bulk (SB). A network including the most relevant molecules and complexes interacting with *Psmc3* (26S proteasome regulatory subunit 6A) is shown. Figure based on Ingenuity Pathway Analysis (IPA) graphs, Qiagen.

In B16F10 miR‐666‐3p^+^ SB cells, antigen processing and presentation were not among the pathways predicted to be strongly dysregulated (see Section 3.3.2). *H* *2‐K^b^* (MHC of the mouse, class I isotype molecule ‘K’ of the ‘b’ haplotype) was the only molecule in the *Antigen Presentation Pathway* whose expression was directly modulated to a significant extent (log_2_(FC) = 1.450, *p*‐value = 1.1 × 10^−04^), leading to a predicted activation of this pathway with IPA (Figure 6). Network analyses of the antigen processing and presentation pathways and their components (including *Psmc* molecules) were performed with IPA. Most of the proteins constituting the proteasome and the immunoproteasome were either downregulated (green) or predicted to be downregulated (blue), but did not lead to a predicted inhibition of antigen presentation, which was actually activated (*p*‐value = 5.2 × 10^−06^, orange) due to a concomitant *H2‐K^b^* upregulation (Figure 6). When focusing on functions regulated by the *Psmc* cluster, *Cell Survival* and *Cell Viability of Tumour Cell Lines* were predicted to be inhibited by miR‐666‐3p overexpression, while *Cell Death of Cancer Cells* and *Necrosis* were predicted to be activated (all with *p*‐values < .001) (Figure 6). These predictions are in line with the observed increase in the susceptibility to CTL‐mediated killing in this cell line. A network with *Psmc3* as a central node was generated, showing that although *Mdm2, Ndufa1* and *Trp53* were connected to *Psmc3*, these molecules were not significantly regulated by mmu‐miR‐666‐3p (Figure 7). In this network, the most strongly and significantly dysregulated molecules were the ribonuclease subunit *Rnaseh2c* (log_2_(FC) = −1.398), *Psmc3ip* (PSMC3 interacting protein, log_2_(FC) = −1.337), which directly interact with *Psmc3* in the network, and the proteasome subunit *Psmb7* (log_2_(FC) = −1.311). Furthermore, *Psmc3* itself was only moderately dysregulated (log_2_(FC) = −0.754) (all *p*‐values < .01, in green) (Figure 7). With B16F10 miR‐320a‐3p^+^ SB cells, these analyses led to very similar results (Figure S4).

In contrast, expression of *H2‐K^b^* was significantly downregulated in B16F10 miR‐7037‐5p^+^ SB cells (log_2_(FC) = –1.111, *p*‐value = 1.4 × 10^−05^), leading to a predicted inhibition of the *Antigen Presentation Pathway* without any strong dysregulation of the *Psmc* cluster components (including *Psmc3ip*) (log_2_(FC) < |1| in all of them) (not shown). As in the other two cell lines, antigen presentation and processing were not among the strongly dysregulated pathways (see Section 3.3.2).

In conclusion, the observed downregulation of the proteasome components and associated molecules, the altered associated functions identified with IPA (e.g., *Cell Death* and *Survival*) and the predicted increase in antigen presentation due to *H2‐K^b^* upregulation go in line with the functional effects observed in mmu‐miR‐666‐3p‐ and hsa‐miR‐320a‐3p‐, but not in mmu‐miR‐7037‐5p‐overexpressing melanoma cells. However, antigen processing and presentation pathways were not among the strongly dysregulated pathways described in the following section. Therefore, it is not expected that they play a critical role in the context of the other strongly impacted pathways.

#### 3.3.2 Dysregulated pathways and functions

To determine the miRNA‐controlled dysregulated pathways and functions, the RNA‐seq data of each selected transductant were subjected to IPA individual *Core Analyses*.

**FIGURE 8:**
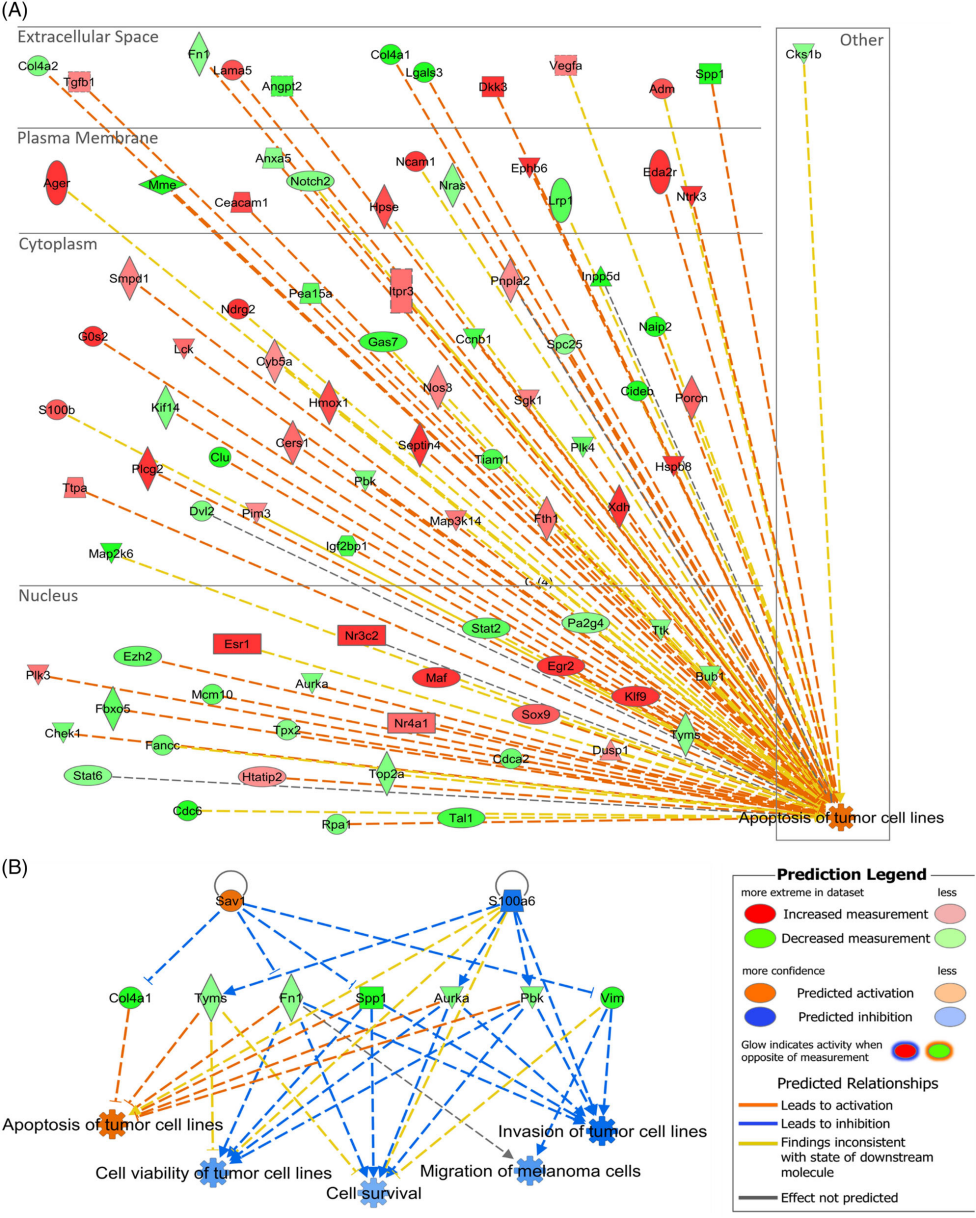
Dysregulated *Apoptosis of Tumour Cell Lines* function and *Regulator Effects* network in B16F10 miR‐7037‐5p^+^ sorted bulk (SB) cells. (A) All the molecules affecting the *Apoptosis of Tumour Cell Lines* function and surpassing the log_2_[fold change (FC)] = |1| and *p*‐value = .001 cut‐offs are depicted using a subcellular location layout. (B) The most relevant network obtained with the *Regulator Effects* Ingenuity Pathway Analysis (IPA) function is depicted (*p*‐values for shown annotations < .001). Figure based on IPA graphs, Qiagen.

In the B16F10 miR‐7037‐5p^+^ SB cell line, the most downregulated *Canonical Pathway* with a *Z*‐score = –1.897 was *Mitogen Activated Protein Kinases (ERK/MAPK) Signalling* (*p*‐value = .02) (Figure S5). This was followed by downregulation of the *Aryl Hydrocarbon Receptor Signalling* (*Z*‐score = –1.667), *Ultraviolet A* *(* *UVA) radiation* *‐induced MAPK Signalling* (*Z*‐score = –1.633), *14‐3‐3 Proteins‐Mediated Signalling* (*Z*‐score = –1.342) and *Wnt/Ca^+^ Pathway* (*Z*‐score = –1.342) (*p*‐values < .05), all consistent with decreased cell survival. Regarding the *Diseases and Biological Functions* influenced by the dysregulated molecules in B16F10 miR‐7037‐5p^+^ SB cells, the category *Cell Death and Survival* (*p*‐value range = 2.7 × 10^−08^ to 8.8 × 10^−05^) was the most relevant annotation predicted to be significantly deregulated. Within it, the two most activated functions were *Apoptosis of Tumour Cell Lines* and *Apoptosis* (*Z*‐scores = 2.185 and 2.131, respectively), and the most inhibited function was *Cell Viability of Tumour Cell Lines* (*Z*‐score = –1.909) (all *p*‐values < .001; Table S5). These results are in line with the observed increased susceptibility to CTL‐mediated killing. Numerous genes strongly dysregulated in the B16F10 miR‐7037‐5p^+^ SB cell line influence the predicted activation of *Apoptosis of Tumour Cells Lines* (Figure 8A), as well as the predicted downregulation of *Cell Viability of Tumour Cell Lines* (Table S5). By applying the *Regulator Effects* IPA function, the molecules dysregulated in our dataset were linked to upstream regulators and downstream affected functions, allowing the detection of key molecules shared by the most relevant functions. The selected network containing the most relevant diseases and functions is shown in Figure 8B. Here, *Fn1, Spp1, Aurka* and *Pbk* appear as putative key players for the effects mediated by mmu‐miR‐7037‐5p, as they participate in the predicted regulation of the majority of the depicted functions.

The same IPA individual *Core Analyses* with B16F10 miR‐666‐3p^+^ SB and B16F10 miR‐320a‐3p^+^ SB cell lines showed that a downregulated *NER* was the top‐ranked *Canonical Pathway*. This and other deregulated pathways in these two cell lines, as well as the generated *Regulator Effects* networks and predicted altered functions are in line with increased tumour cell death (Figures S6–S9 and Tables S6 and S7).

The general activation of pathways leading to increased cell death observed in the IPA software analyses was further supported by in vitro assays in which different doses of γ‐irradiation were applied to these cell lines. A tendency towards enhanced vulnerability was observed in B16F10 miR‐320a‐3p^+^ SB cells and to a lesser extent in the B16F10 miR‐666‐3p^+^ SB cell line in response to radiation, resulting in increased proportions of apoptotic and necrotic cells relative to the control line (Figure S10A–C). However, B16F10 miR‐7037‐5p^+^ SB cells showed no enhanced frequencies of apoptotic or necrotic cells following irradiation in comparison to the control cell line.

Overall, these IPA *Core Analyses* enabled the association of each overexpressed miRNA with dysregulated pathways, functions and molecules that could be responsible for the observed functional effects.

Further results are described in Supporting Information.

### 3.4 Correlation of miRNA expression with disease stage and survival in human melanoma

**FIGURE 9:**
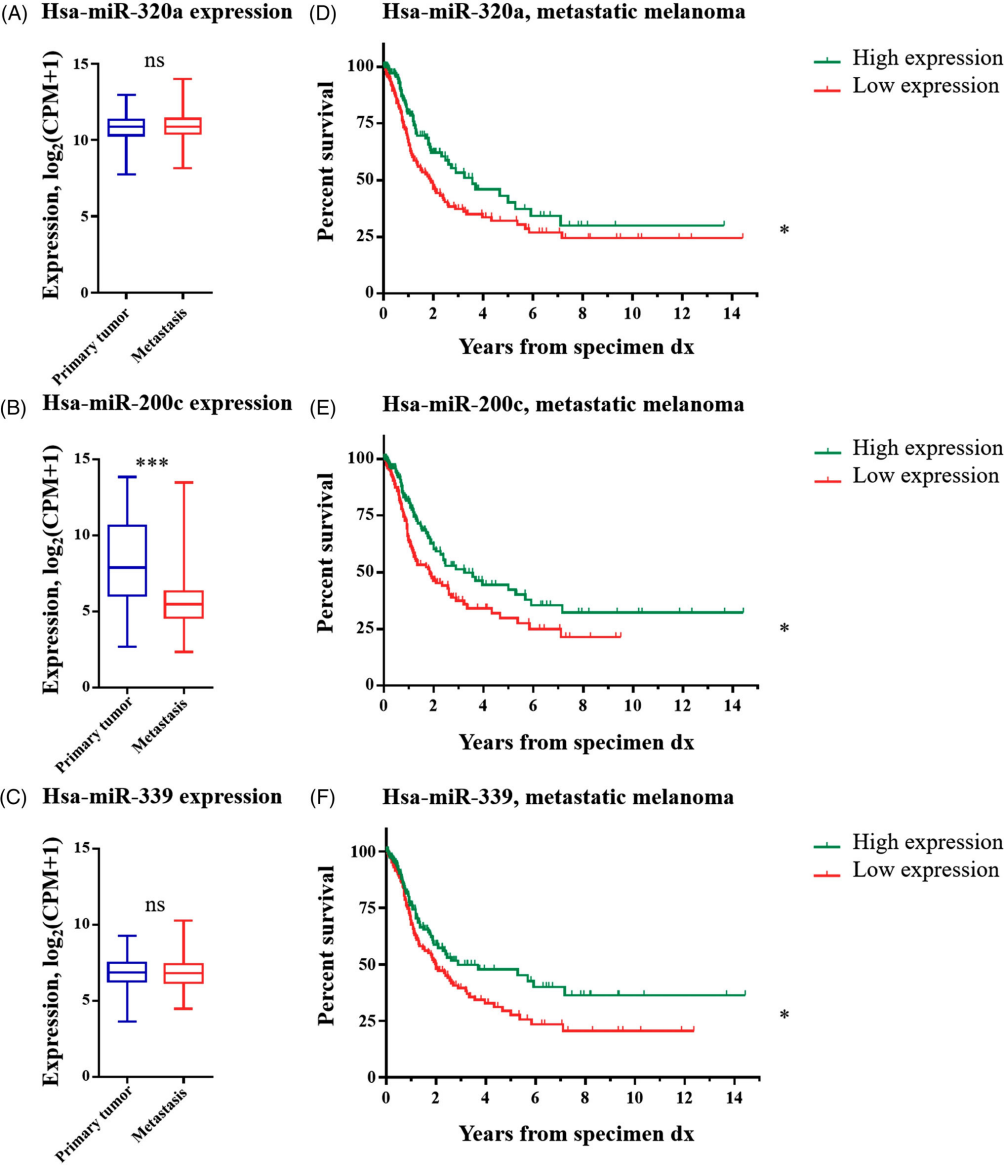
Correlation of microRNA (miRNA) expression with disease stage and survival in human melanoma. (A–C) The log_2_[counts per million (CPM) + 1] expression values of the indicated miRNAs in human melanoma samples acquired from The Cancer Genome Atlas (TCGA) were divided into the two compared groups (primary melanoma: 99 patients, metastatic melanoma: 353 patients). The box plots show the median of each group and the whiskers are the maximum and minimum values. Unpaired two‐sided *t*‐tests and Mann–Whitney tests were calculated for each comparison and the significance levels were the same with both tests. (D–F) Using 351 metastatic melanoma samples, the survival of two groups of patients divided according to the levels of expression of each miRNA are compared. Kaplan–Meier survival curves were generated and Log‐rank (Mantel–Cox) tests were performed in each case. Statistical tests significance with *p*‐values adjusted for multiple testing using the Holm method: ^*^ *p* ≤ .05, ^***^ *p* ≤ .001, ns: not significant. dx: diagnosis. Human miRNAs shown here are conserved in mice.

Among the top‐ranked miRNAs listed in Table S2, hsa‐miR‐320a‐3p, hsa‐miR‐200c‐3p and hsa‐miR‐339‐5p are conserved between mice and humans as determined using miRBase.^44^ Based on TCGA data from melanoma patients,^45^ the correlations between the expression of these miRNAs and disease stage as well as survival of cancer patients were determined. Since the identified miRNAs had increased the susceptibility to CTL‐mediated killing in our functional experiments, a lower expression of these miRNAs in advanced metastatic melanoma was postulated. Indeed, a significantly lower expression of hsa‐miR‐200c was found in metastasis samples compared to primary tumours (Figure 9B), whereas the expression of hsa‐miR‐320a and hsa‐miR‐339 showed no statistical differences between these two groups (Figure 9A,C). More importantly, high expression of all analysed miRNAs correlated with a statistically significant survival benefit to melanoma patients, indicating that these miRNAs may also exert tumour‐suppressive effects in humans (Figure 9D–F).

## 4 DISCUSSION

Although some studies have already investigated how alterations in melanoma cells affect their susceptibility to CTL effector functions,^46^ screenings focused on the impact of overexpressed miRNAs in melanoma cells on CTL‐mediated killing are still missing, leaving an untapped potential for the discovery of miRNAs for the development of new miRNA‐based cancer therapies. Our comprehensive miRNA screening identified for the first time a set of miRNAs that significantly increased T cell‐mediated cytotoxicity of melanoma cells. In support to our findings, miR‐320a,^47^, ^48^ miR‐200c,^49^, ^50^ miR‐326^51^, ^52^ and miR‐339^53^, ^54^ had already been described as tumour‐suppressive in different cancers, and miR‐320a,^55^ miR‐200c^56^ and miR‐326^57^ were found to be tumour‐suppressive in melanoma. Regarding miR‐339, only the 3p strand had been shown to have an onco‐suppressive effect in melanoma,^20^ but not the 5p strand, which was selected in this study. Interestingly, miR‐320a was found to modulate PD‐L1 expression in malignant mesothelioma, which may contribute to immune evasion in these tumours.^58^ However, our RNA‐seq analysis of the hsa‐miR‐320a‐3p‐overexpressing melanoma cell line showed no significant dysregulation of PD‐L1 (*p*‐value > .1). Another study used nanoparticles to treat colon carcinoma, combining a BRAF inhibitor with miR‐200c, which can downregulate PD‐L1 expression and participate in the enhancement of the anti‐tumour immune response.^59^ In our ELISpot assays, we showed that neither hsa‐miR‐320a‐3p‐ nor hsa‐miR‐200c‐3p‐transfected melanoma cells led to an increased T cell IFNγ secretion, suggesting that a possible PD‐L1 downregulation is probably not the cause of the increased cytotoxicity. Lastly, for miR‐666 (specifically the 3p strand), miR‐7082 and miR‐7037, no publications on the role of these miRNAs in the modulation of tumour cell susceptibility to CTL lysis are available to date, underlining the novelty of our results.

With the goal of finding common miRNA targets that could be involved in the effects observed in the screen, a separate in silico screen‐wide miRNA target analysis was performed, allowing the selection of relevant predicted target genes. Interestingly, the best ranked selected genes were not explicitly related to antigen presentation or CTL‐mediated cell death. However, they could affect mechanisms that have not yet been found to be connected to the observed functional effects, a result that was also seen in previous studies.^46^ The siRNA‐mediated downregulation of two predicted targets *Psmc3* and *Ndufa1* (each a common predicted target of six miRNAs that increased CTL cytotoxicity in the screen, Table S10) had significant effects on the CTL‐mediated killing, indicating that these molecules could play a key role in the mechanisms involved (Figure 4).

Considering our translational goal of pinpointing the most effective miRNA candidates, we focused on the three best performing miRNA‐overexpressing cell lines to gain a deeper understanding of the molecular mechanisms underlying the observed increased cytotoxicity. RNA‐seq and IPA software analyses revealed that each tested miRNA modulated specific pathways, but there were also some commonalities. Based on the dysregulation of the expression of various genes, *NER* was predicted to be the top‐ranked downregulated *Canonical Pathway* in B16F10 miR‐320a‐3p^+^ SB and B16F10 miR‐666‐3p^+^ SB cell lines, leading to the activation of functions related to cell death accompanied by an inhibition of functions related to cell survival and viability. An analysis using IPA tools was used to predict the impact on cell functions of dysregulated genes in the most relevant pathways. In the case of *NER* in B16F10 miR‐320a‐3p^+^ SB cells, inhibition of almost all the steps in the repair mechanism was predicted (Figure S8). This repair mechanism had been shown to be deficient in melanoma and participates in repair of ultraviolet light‐induced DNA damage, which is linked to melanoma development.^60^ *NER* is involved in deciding cell fate by leading to repair initiation or apoptosis. In this pathway's analysis with IPA, we saw that while *Cell Viability of Tumour Cell Lines* and *Cell Survival* were predicted to be inhibited, *Cell Death of Tumour Cell lines* and *Apoptosis* were predicted to be activated. *NER* in B16F10 miR‐666‐3p^+^ SB led to the same predictions with the exception that *Apoptosis* was only mildly activated (Figure S6). All these predictions go in line with an increased susceptibility to cell death. Furthermore, antigen presentation was predicted to be upregulated in B16F10 miR‐320a‐3p^+^ SB and B16F10 miR‐666‐3p^+^ SB cells and a general inhibition of the proteasome, including the downregulation of at least one molecule within the *Psmc* cluster, was also observed. This cluster's downregulation was shown to be causally connected to a predicted activation of *Cell Death of Tumour Cells* and a predicted inhibition of *Cell Viability of Tumour Cell Lines*, showing a clear association with the observed functional effects.

*Psmc3*, which plays an important role in proteasome functions and whose siRNA knockdown significantly increased melanoma cells’ susceptibility to CTL‐mediated killing (Figure 4), is interconnected in the above‐mentioned networks. In B16F10 miR‐320a‐3p^+^ SB and B16F10 miR‐666‐3p^+^ SB cell lines, *Psmc3ip* (whose protein was shown to interact with human PSMC3^61^) was much more downregulated than *Psmc3* and more relevant in the predictions, therefore indicating that PSMC3IP might play a role in the effects observed. We hypothesise that the inhibition of the proteasome function achieved by the downregulation of *Psmc3*, *Psmc3ip* or possibly further proteasome regulatory subunits could drastically affect processes such as apoptosis and cellular stress,^62^ leading to cell destabilisation. The accumulation of damaged proteins could make the cell more susceptible to different kinds of stress, including death stimuli from CTLs. This mechanism would only be relevant if the proteasome was functional enough to lead to sufficient antigen presentation. Considering the increased killing observed in the cytotoxicity assays with B16F10 miR‐320a‐3p^+^ SB and B16F10 miR‐666‐3p^+^ SB cell lines, as well as with the siRNA‐mediated knockdown of *Psmc3*, it is clear that the antigen processing and presentation mechanisms are not abrogated in these cases. Lastly, PSMC3 was found to be an unfavorable prognostic marker in human renal cancer,^63^ further supporting its possible involvement in cancer development and immune escape.

Although the *Canonical Pathways* analysis with B16F10 miR‐7037‐5p^+^ SB cells did not uncover many commonalities with the two cell lines described above, the most dysregulated pathways in this cell line were also correlated with decreased cell survival. The most downregulated canonical pathway was *ERK/MAPK Signalling*, which regulates various cell processes including cell growth, proliferation and survival, and has been shown to play a key role in different tumours, including melanoma.^64^ Hence, it is logical to observe increased tumour cell susceptibility to cell death if this pathway is downregulated. When analysing *ERK/MAPK Signalling* based on all dysregulated molecules in B16F10 miR‐7037‐5p^+^ SB cells using IPA predictions, *Cell Survival* was shown to be inhibited (Figure S5), which goes in line with an increase in cell death.

The IPA *Diseases and Biological Functions* analysis uncovered similar profiles with all three analysed cell lines. In general, apoptosis was upregulated, while survival‐ and viability‐related functions were downregulated, and these predictions were based on tens to hundreds of significantly dysregulated molecules (Tables S5–S7 and Figure 8A).

In conclusion, the IPA *Core analyses* showed us many different mechanisms that could be synergistically work towards the functional effects observed. Interestingly, although antigen presentation was predicted to be upregulated by hsa‐miR‐320a‐3p and mmu‐miR‐666‐3p, this pathway was not among the ones predicted to be strongly dysregulated; therefore, it is not expected to play a significant role in the context of all the other deregulated pathways. Going beyond the *Psmc3*‐related networks, we see many deregulated *Biological Functions* that are directly related to cell death, and *Canonical Pathways*, whose predicted dysregulation is directly correlated to decreased cell survival. Taking these results into account, it may be hypothesised that the dysregulation of sizeable groups of molecules by each miRNA makes the tumour cells more susceptible to death‐inducing stimuli (e.g., T cell‐mediated tumour cell killing), without significantly affecting their growth rate or survival in the absence of stressors. As these mechanisms are not specific to T‐cell‐mediated killing, it would also be interesting to test the effects of these miRNAs under other stressful stimuli that induce cell death. This was assessed experimentally using γ‐irradiation, and the cell lines overexpressing hsa‐miR‐320a‐3p and mmu‐miR‐666‐3p showed a tendency towards enhanced vulnerability to this stressor.

Furthermore, the *Regulator Effects* function enabled us to uncover dysregulated molecules modulating various functions at the same time and, therefore, possibly playing an important role in the functional effects observed. Among these genes, some were relevant in more than one cell line, for example, *Spp1, Aurka* and *Pbk* were relevant in both B16F10 miR‐7037‐5p^+^ SB and B16F10 miR‐666‐3p^+^ SB (Table S8). Further in‐depth studies of these molecules could shed more light on the leading mechanisms through which these miRNAs act. However, the massive number of genes significantly regulated by each tested miRNA (Table S4) (which is a defining characteristic of many miRNA species^17^, ^18^) and the numerous molecules found to affect the relevant biological functions uncovered support the conclusion that only a few dysregulated molecules might not be completely responsible for the effects described. Biochemical techniques that detect large‐scale miRNA–mRNA interactions, as well as mass spectrometry proteomic analyses might provide additional evidence of the dysregulations already observed at the RNA level,^18^ thereby complementing the results of our study.

Interestingly, though mediating enhanced CTL lysis of transfected target cells, the validated miRNAs failed to induce increased IFNγ secretion by co‐cultured CTLs, except in the case of mmu‐miR‐7082‐5p (Figure 2B). This is compatible with the IPA software analyses of the RNA‐seq data discussed above, as the uncovered putative mechanisms do not require increases in CTL activity to explain enhanced target cell death. In fact, it was shown that cytokine secretion levels and cytotoxic activity of CTLs do not always correlate.^65^, ^66^ These studies suggest that a disrupted CTL effector function or caspase activity in the target cells could lead to higher IFNγ secretion accompanied by a lower killing efficiency. Hence, we hypothesise that alterations triggered in tumour cells by the miRNAs may lead to more efficient and faster CTL‐mediated induction of target cell death, a quicker T cell detachment, and possibly even lower levels of IFNγ secretion. This concept is in line with our IPA software analyses, in which all the tested miRNA‐overexpressing cell lines showed predicted activation of the apoptotic pathway.

The sensitising effects of the selected miRNAs on CTL‐mediated melanoma cell eradication were validated in vitro on the murine melanoma cell line B16F10. The generation of tumour cell lines with stable miRNA overexpression in other tumour entities might offer a tool to verify the miRNA‐mediated effects on CTL susceptibility in further cancer types, and they could also be tested in in vivo murine models. Interestingly, the TCGA data analysis of melanoma patients revealed that the conserved miRNA hsa‐miR‐200c‐3p showed a significantly decreased expression in metastatic versus primary melanoma, suggesting an onco‐suppressive activity. Supporting our findings, similar results were shown in another study that reported that miR‐200c was downregulated in primary and metastatic melanoma tissues versus nevi samples and that it was also decreased in human metastatic melanoma cell lines compared to primary melanoma cell lines, indicating that the levels of this miRNA are related to melanoma progression.^56^ Furthermore, we noted that high expression levels of the three conserved miRNAs in metastatic melanoma patients correlated with statistically significant increases in survival, supporting the notion that these miRNAs may play an onco‐suppressive role. As this study was performed using murine cell lines and most of the selected miRNAs are not conserved in humans, further experiments that go beyond the scope of this study would increase the translational potential of our miRNA candidates. The generation of human melanoma cell lines stably expressing the miRNA candidates, their validation with CTL killing assays and subsequent RNA‐seq analyses would uncover the genes modulated in human cells. An analysis to assess the correlation of the expression of these genes and the conserved miRNA expression in melanoma patients, as well as the use of human xenograft models in vivo, would also complement this study.

Although the field of miRNA‐based cancer therapy is still in its infancy, it holds great promise. One main advantage of such approaches is the fact that most miRNAs do not target a single molecule (such as siRNAs or small molecules) but, instead, sizeable clusters of them,^17^, ^18^ thereby counteracting the development of therapy resistance. Furthermore, the capacity of the presented miRNAs to enhance the susceptibility of tumour cells to CTL lysis without inducing cell death per se could minimise possible damage to healthy tissues and reduce associated side effects if used as a treatment.

## 5 CONCLUSIONS

In conclusion, our study uncovers a group of miRNAs that significantly increase susceptibility of melanoma cells to CTL lysis and determines miRNA targets and networks potentially involved in these effects. Furthermore, we found correlations between expression of a subset of these miRNAs and clinical parameters in melanoma patients. The presented miRNAs might help to improve the development of both classical chemotherapy approaches, as well as novel immunotherapeutic treatments against melanoma and other tumours.

## CONFLICTS OF INTEREST

Antonino A. Pane is currently affiliated with Immatics Biotechnologies; however, this study was conducted while affiliated with the DKFZ. The remaining authors declare they have no conflicts of interest.

## Supporting information

Supporting Information
