# Supplementary material for: MicroRNAs affecting the susceptibility of melanoma cells to CD8+ T cell‐mediated cytolysis
Source: Clin Transl Med. 2023 Jan 30;13(2):e1186. doi: 10.1002/ctm2.1186 (PMC9887093; doi:10.1002/ctm2.1186)
Supplement: Supplementary file 1 — Supporting Information [file CTM2-13-e1186-s001.pdf]

Supporting information to the to the manuscript:

miRNAs affecting the susceptibility of melanoma cells to CD8+ T cell-mediated cytolysis

Antonino A. Pane[1,2,†], Theresa Kordaß[1,2,§], Agnes Hotz-Wagenblatt[3], Elke Dickes[1], Annette Kopp-Schneider[4], Rainer Will[5], Barbara Seliger[6], Wolfram Osen[1], Stefan B. Eichmüller[1*]

[1]Research Group GMP & T Cell Therapy, German Cancer Research Center (DKFZ), Heidelberg, Germany

[2]Faculty of Biosciences, University Heidelberg, Heidelberg, Germany

[3]Omics IT and Data Management Core Facility, DKFZ, Heidelberg, Germany

[4]Biostatistics Department, DKFZ, Heidelberg, Germany

[5]Core Facility Cellular Tools, DKFZ, Heidelberg, Germany

[6]Institute of Medical Immunology, Martin-Luther-University Halle-Wittenberg, Halle/Saale, Germany

[†]Current affiliation: Immatics Biotechnologies GmbH, Tübingen, Germany

[§]Section Multiple Myeloma, Internal Medicine V, University Clinic Heidelberg, 69120 Heidelberg, Germany

Methods

Cell culture and viral transduction of tumor cell lines

B16F10 cells obtained from ATCC were cultured in PS (penicillin, streptomycin) RPMI medium containing 10% (v/v) FBS + 100 Units/mL penicillin + 100 µg/mL streptomycin at 37 °C/5% CO_2. These cells were transduced with retroviruses expressing red firefly luciferase (pBabe-Puro red firefly) or lentiviruses expressing wildtype OVA (rwpLX305_Ovalbumin_IRES_Neo). B16F10 Luci$^+$ OVA$^+$ cells were transduced with custom made lentiviruses (Applied Biological Materials Inc.) that included the sequences for the mature strands of the miRNAs (and for a *C. elegans* miRNA: CGGUACGAUCGCGGCGGGAUAUC for B16F10 miR-Neg. Control) co-expressed with green fluorescent protein (GFP) under the same cytomegalovirus promoter. For each cell line, three clones with different GFP expression levels were established using FACS single-cell sorting. Sorted GFP$^+$ bulk cultures (SB cell lines) were obtained using flow cytometry upon gating on cells with a high to medium GFP expression level using a FACSAria Fusion cell sorter (BD). B16F10 Luci$^+$ cells were cultured in PS RPMI medium supplemented with puromycin (1 µg/mL), whereas B16F10 Luci$^+$ OVA$^+$ cells were cultured in this same medium containing geneticin (1 mg/mL) and puromycin (1 µg/mL). B16F10 Luci$^+$ OVA$^+$ cells transduced with miRNA encoding lentivirus were expanded in PS RPMI medium containing geneticin (1 mg/mL), puromycin (1 µg/mL) and hygromycin (100 µL/mL). RMA TRP-2 cells[1] were cultured in PS RPMI medium with Geneticin (0.8 mg/mL) (***Table S13***). Functional assays were performed in RPMI medium with 10% (v/v) FBS and without antibiotics.

T cell culture and re-stimulation

The TRP-2 specific CTLs[1] (line 5a, clone Nβ) were cultured in complete T cell medium at 37°C and 5% CO_2 in 24-well plates (medium composition in ***Table S13***). A clone from this CTL line was obtained by limiting dilution and used in the assays, and the cells were expanded by repeated *in vitro* weekly re-stimulations. For this process, spleens were removed from C57BL/6 mice, mashed through a 70 µm filter (EASYstrainer, Greiner) and collected in a 50 mL tube with PBS. After a 5-minute centrifugation at 300 g, the supernatant was discarded and 4 mL/spleen of ammonium-chloride-potassium (ACK) Lysing Buffer (Gibco) was added. After 90 seconds of resuspension, the cells were washed with PBS, they were resuspended in PS RPMI medium and then irradiated with 33 Gy. Moreover, RMA TRP-2 cells were harvested in PS RPMI medium and irradiated with 200 Gy. Finally, 5 x 10^6 splenocytes and 2 x 10^5 RMA TRP-2 cells were added to each well of the 24-well plates with freshly seeded CTLs.

Transfection of siRNAs and miRNAs

The Metafectene SI^+ transfection reagent (Biontex) was used following the manufacturer's instructions. Two thousand tumor cells/well were seeded on top of the si/miRNA - reagent mixture in a flat bottom 96-well plate (for a final concentration of 50-100 nM indicated in each case) and then cultured for three days. Pools of 30 siRNAs targeting each transcript (siPOOLs, siTOOLs Biotech) were used in the knockdown experiments, and siRNA AllStars (Qiagen) was used as negative control. Regarding the miRNAs, the negative control miRNA ctrl. Pool 1 was generated by mixing 171 miRNAs from the miRNA library in equal amounts. The following miRNAs were used for the quality control of the screen: miRNA Mimic Non-Targeting Control 1 (Active Motif-SwitchGear Genomics), mmu-miR-7025-5p, mmu-miR-6969-5p and mmu-miR-6926-3p. MirVana miRNA mimics (Thermo Fisher Scientific) from the library were used in the screen and initial validation assays, followed by application of miRIDIAN microRNA mimics (Dharmacon) (***Fig. 2 A, B***).

RT-qPCR for mRNA quantification

RNA was extracted with the RNeasy Plus Micro Kit (Qiagen), the nucleic acids were quantified with the NanoDrop (Thermo Scientific) and the Transcriptor First Strand cDNA Synthesis Kit (Roche) was used to obtain cDNA, according to the respective manufacturer's instructions. The PCR mix contained the following components per well: 10 µL of master mix (PowerUp SYBR Green PCR Master Mix, Thermo Fisher Scientific), 0.4 µL of each primer (10 µM each), 7.2 µL of RNase/DNAse free water and 2 µL/well of diluted cDNA. The qPCRs were run in a 7300 Real Time PCR System thermocycler (Applied Biosciences) using the following protocol: Step 1 (50 °C for 2 minutes, 1 cycle), step 2 (95 °C for 2 minutes, 1 cycle), step 3 (95 °C for 15 seconds + 60 °C for 1 minute + 72 °C for 30 seconds, 40 cycles) and step 4 (95 °C for 15 seconds + 60 °C for 1 minute + 95 °C for 15 seconds + 60 °C for 15 seconds, 1 cycle). Analysis of ß-actin was used as the housekeeping gene. To calculate the relative mRNA expression, the $2^{(-\Delta\Delta Ct)}$ method was used generating fold change (FC) values vs. control, which were employed to determine the percentage of knockdown with the following formula: Knockdown (%) = (1 - FC) x 100%. Primers for RT-qPCR assays are given in ***Table S14***.

RT-qPCR for miRNA quantification using TaqMan™ assays

The miRNA extraction was performed using the miRNeasy mini Kit (Qiagen), the RNA was quantified with the Qubit device (Invitrogen) and its purity was assessed using the NanoDrop device (Thermo scientific) following the respective manufacturer's instructions. TaqMan advanced miRNA assays with the TaqMan Fast Advanced Master Mix (Thermo Fisher Scientific) were used following the

manufacturer's protocol and the housekeeping miRNA employed was mmu-miR-25-3p. The qPCRs were run in a 7300 Real Time PCR System thermocycler (Applied Biosciences) with the instrument settings in the TaqMan advanced miRNA assays manufacturer's protocols. The relative miRNA expression in each cell line in comparison with parental was calculated using the $2^{(-\Delta\Delta Ct)}$ method to obtain FC.

IFNγ ELISpot assay

The ELISpot plate (Millipore) was pre-treated with 20 µL/well of 80% ethanol and coated with a rat anti-mouse IFN-γ antibody (Ab) (BD) (5 µg/mL). After incubation at 4°C for 24 hours, the plate was blocked with medium containing 10% FBS (v/v) for one hour at 37°C, and then the CTLs (12500/well) and the previously transfected B16F10 Luci$^+$ cells (12500/well) were added. After 18 hours at 37 °C/5% CO_2, the plate was washed with a 0.5% (v/v) tween 20 in PBS solution and, afterwards, with PBS. Next, after a one-hour incubation at 4 °C with a biotin-conjugated rat anti-mouse IFN-γ Ab (BD) (1 µg/mL), the plate was washed with PBS and incubated with 100 µL/well of a 500-fold dilution of Alkaline Phosphatase Streptavidin (BD) in PBS for 30 minutes at RT in the dark. After washing, 100 µL/well of BCIP/NBT Liquid Substrate System (Sigma) were added and the reaction was stopped with distilled water. A Cellular Technology Limited ELISpot reader was used to count the spots and GraphPad Prism software was employed for the statistical analysis. Four replicates of each condition per plate were used. If wells were discarded due to a contamination or technical error, three wells were used for calculation.

Impedance based cytotoxicity assay

The xCELLigence RTCA Multi Plate instrument (ACEA Biosciences) was used to measure and monitor CTL-mediated target cell killing in real time. The miRNA-transduced B16F10 Luci$^+$ OVA$^+$ cells were cultured in an E-Plate VIEW 96 PET (ACEA Biosciences; 8000 cells/well) for 26 hours at 37°C/5% CO_2 followed by addition of 36000 TRP-2 specific CTLs/well. The RTCA Software 2.0 (ACEA Biosciences) measured the cell index (proportional to cell amount) every 15 minutes. The cell index was normalized to the time point of CTL addition and average curves were generated. An R code was created to determine the statistical significance of the difference between the magnitudes of cytotoxicity obtained with the control cell line (B16F10 miR-Neg. Control, clone M) vs. the miRNA-transduced cell lines using areas beneath the curves and *t*-tests. The data was exported from the RTCA software and plotted using Excel. Four replicates of each condition per plate were used. If wells were discarded due to a contamination or technical error, three wells were used for calculation.

RNA-seq experiment and raw data analysis

Total RNA was extracted from three independent samples from each miRNA expressing cell line using the RNeasy Plus Mini kit (Qiagen). The purity and the concentration of the RNA was assessed using the NanoDrop device (Thermo Scientific) and the samples were processed for a HiSeq 4000 Single-Read 50bp sequencing. Low quality bases were removed with Fastq_quality_filter from the FASTX Toolkit 0.0.13[2] with 90% of the read needing a quality phred score > 20. Homertools 4.7[3] were used for PolyA-tail trimming, and reads with a length < 17 were removed. Genomic mapping was performed with STAR 2.3[4] for the filtered reads with mouse genome 38. For quality checking, PicardTools 1.78 CollectRNASeqMetrics[5] was performed on the mapped reads. Count data was generated by FeatureCounts v1.4.5-p1[6] with parameters minReadOverlap 3 -T 3 -M -s 2 using the gencode.vM23.annotation.gtf[7] file for annotation. After this, the FC between each miRNA expressing cell line and the control cell line (B16F10 miR-Neg. Control, clone M) was calculated with DESeq2[8]. For these comparisons, the input tables containing the replicates with the compared groups were created by a custom perl script. In the count matrix, rows with an average count number < 10 were removed, then DESeq2 (version 1.4.1) was run with default parameters. The resulting tables were annotated with gene information (gene symbol, gene type) derived from the gencode.vM23.annotation.gtf file.

Analyses using TCGA data

Using the clinical and miRNA expression data of melanoma patients from TCGA (452 samples), the first analysis compared the expression of each miRNA in primary tumor and metastasis. The Log_2(CPM+1) was calculated for each expression value and they were plotted using GraphPad Prism. Unpaired two-sided *t*-tests and Mann-Whitney tests were performed for each comparison. Furthermore, including metastatic melanoma patients that had survival data in TCGA (351 patients), an analysis to find correlations between miRNA expression and survival was performed. Using the median of the Log_2(CPM+1) expression values for each miRNA as a threshold, the patients were sorted into two groups with high and low miRNA expression. GraphPad Prism was used to generate Kaplan-Meier survival curves and to perform Log-rank (Mantel-Cox) tests for each miRNA.

Flow cytometry for detection of viable, apoptotic and necrotic cells after irradiation

$2x10^5$ cells of the SB cell lines (B16F10 miR-Neg. Control SB, B16F10 miR-7037-5p$^+$ SB, B16F10 miR-320a-3p$^+$ SB and B16F10 miR-666-3p$^+$ SB) were cultured per well in 12-well plates in 1 mL RPMI Medium + 10 % FCS w/o antibiotics. After 24 h, cells were treated with various irradiation

doses (0, 5, 10 or 20 Gy) using ^{137}Cs as source of γ-radiation (Gamma Cell 40, Atomic Energy of Canada). Then, 1 mL of fresh RPMI medium was added to each well, and following incubation for 24 h, the proportion of apoptotic, necrotic and live cells was determined using the Annexin V Apoptosis Detection Kit I (BD Biosciences) according to manufacturer's protocol. Briefly, supernatant of wells was collected and cells were detached with 400 µL Accutasee/well (2 min at RT). Harvested cells were centrifuged and washed twice with ice-cold PBS followed by staining with PE-Annexin and 7-AAD in 1X Binding Buffer for 15 min at RT in the dark (200 µL Binding Buffer + 10 µL PE-Annexin + 10 µL 7-AAD per sample). Thereafter, 200 µL 1X Binding buffer was added to each sample and cells were immediately measured by flow cytomery using a Canto flow cytometer (BD). Data analysis was performed with FlowJo software. Gating strategy was based on unstained cells and on single-stained heat killed cells.

Results and Figures

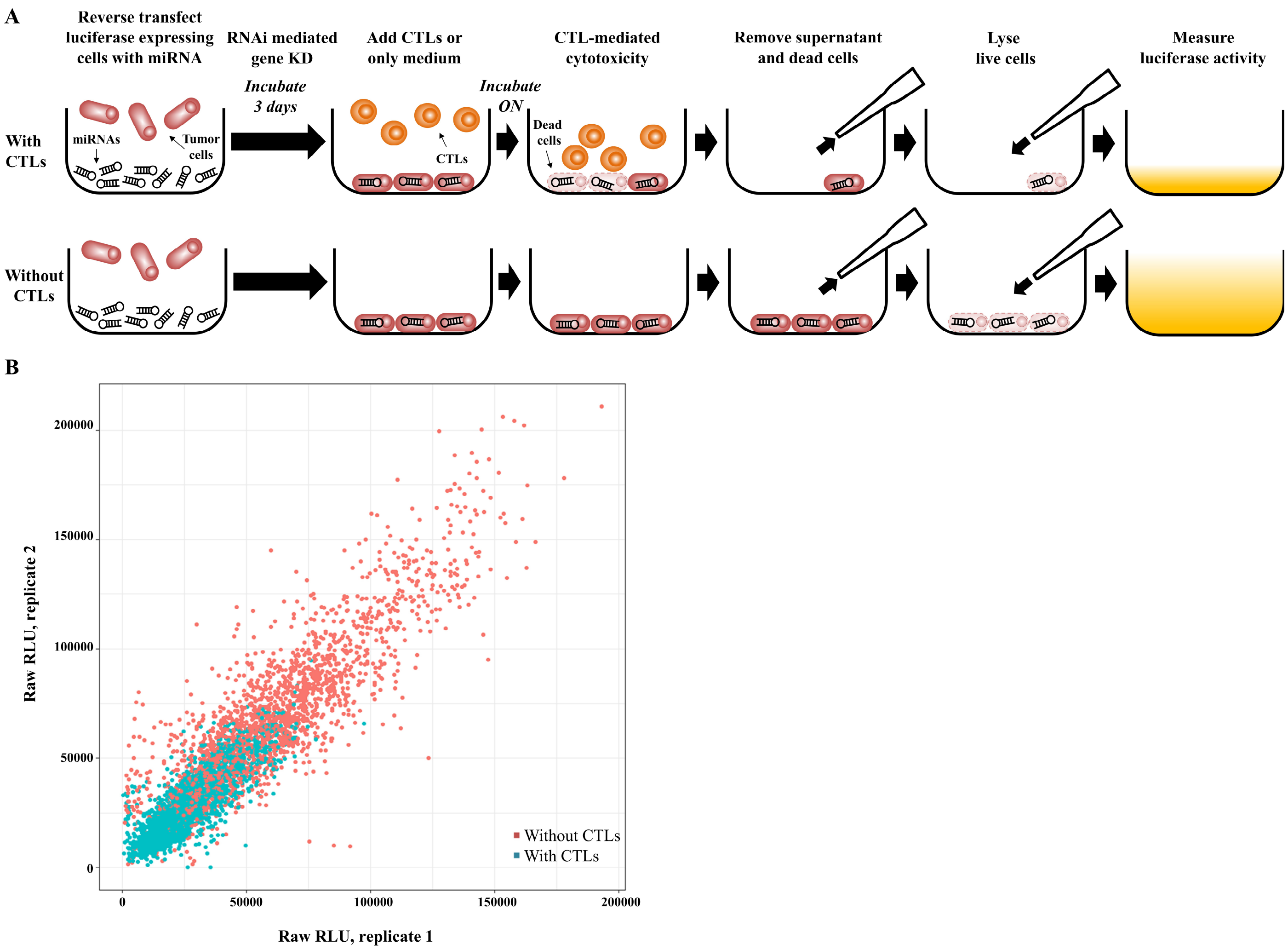

Figure S1: Luciferase assay principle and high-throughput miRNA screen results. A) The different steps of the luciferase assay used in the high-throughput miRNA screen are shown. Based on the assay from Khandelwal N. *et al.*[9]. **B)** The raw relative luminescence units (RLU) values from the luciferase assays, proportional to the amount of live tumor cells in each well, were obtained from both replicates of each assayed miRNA in the whole screen, and are plotted against each other to check for reproducibility. The differences in RLU values between wells treated with (blue) and without (red) CTLs evidence the CTL-mediated killing. CTLs: cytotoxic T lymphocytes, KD: knockdown, ON: overnight.

Supporting information to "Screen-wide *in silico* analysis uncovers predicted miRNA targets affecting CTL-mediated lysis"

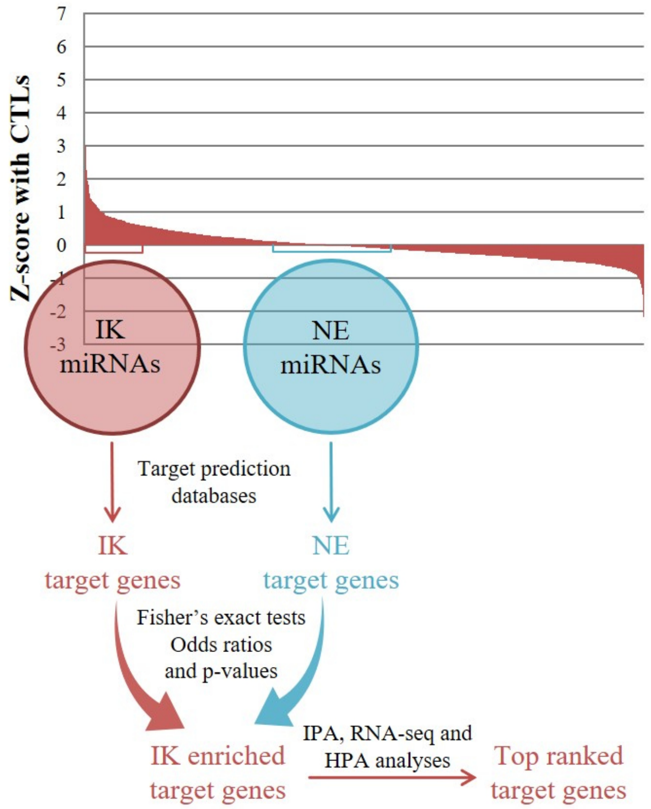

Figure S2: Screen-wide strategy of the *in silico* analysis of the screen results. The scheme shows the multistep *in silico* analysis performed with the results from the screen to select the top ranked predicted miRNA target genes. CTLs: cytotoxic T lymphocytes, IK: increased killing, NE; no effect on killing, IPA: Ingenuity Pathway Analysis, HPA: Human Protein Atlas.

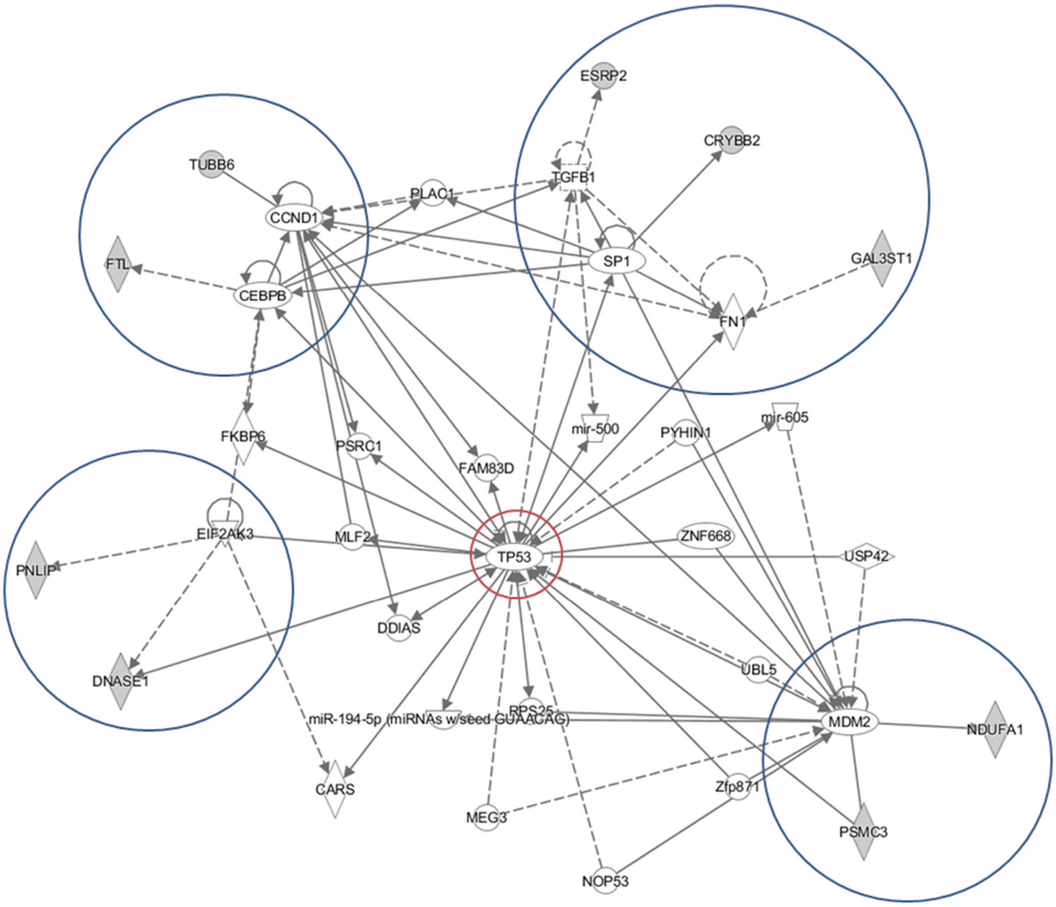

Figure S3: IPA network 2 from the *in silico* analysis of the screen results. The interactions within the network are shown using an organic layout. The closely interacting enriched target molecules that are included (gray filled shapes) are highlighted with blue circles and the proteins of the four top ranked selected molecules (*Ftl1*, *Tubb6*, *Ndufa1* and *Psmc3*) are within them. In this network, TP53 plays a central role (red circle). Figure based on IPA (Ingenuity Pathway Analysis) graphs, Qiagen.

Supporting information to "RNA-seq analysis uncovers pathways, functions and networks dysregulated by the selected miRNAs"

Dysregulated relevant molecules, antigen presentation and proteasome function

In B16F10 miR-320a-3p^{+} SB cells, *H2-K^{b}* was again the only significantly regulated molecule in the *Antigen Presentation Pathway* (log_2(FC) = 1.682, p-value = 2.5x10^{-05}), leading to a predicted increase in MHC class I antigen presentation (***Fig. S4 A***). Network analysis including the antigen processing and presentation pathways and the *Psmc3*-centered network revealed results similar to those seen with B16F10 miR-666-3p^{+} SB cells (***Fig. S4 A, B***), but *Psm*c3 expression was not significantly downregulated in this case (log_2(FC) = -0.532, p-value = 0.1). In the *Psmc3*-centered network, *Rnaseh2c* (log_2(FC) = -1.725), *Psmc3ip* (log_2(FC) = -1.565) and *Psmb7* (log_2(FC) = -1.076) were significantly dysregulated (all p-values < 0.01; ***Fig. S4 B***), which is consistent with the results from B16F10 miR-666-3p^{+} SB cells, further supporting a possible role of these molecules in modulating anti-tumoral immune responses of melanoma cells.

Dysregulated pathways and functions

Performing an IPA individual *Core Analysis* with the data from the B16F10 miR-666-3p^{+} SB cell line revealed *NER* as the top ranked *Canonical Pathway,* which was predicted to be strongly downregulated (Z-score = -3.638, p-value = 6.6x10^{-08}). In this pathway, a strong downregulation of several molecules within its network (log_2(FC) < -1 in more than 10 molecules) led to the predicted downregulation of all the steps in the repair mechanism. This affected relevant functions including *Cell Survival* and *Cell Viability of Tumor Cell Lines*, which showed a strong predicted inhibition, while *Cell Death of Tumor Cell Lines* and *Apoptosis* were predicted to be upregulated (p-values < 0.001; ***Fig. S6***). The most upregulated *Canonical Pathway* was *Sumoylation Pathway* (Z-score = 3.0, p-value = 0.02), affecting, among others, the functions *Apoptosis of Tumor Cells* and *Cell Death of Melanoma Cell Lines.* These functions were predicted to be strongly activated (p-values < 0.001; data not shown), which is in line with the increased susceptibility to CTL-mediated cytotoxicity in the B16F10 miR-666-3p^{+} SB cell line. The *Diseases and Biological Functions* analysis with this cell line showed once again *Cell Death and Survival* (p-value range = 2.7x10^{-08} - 8.8x10^{-05}) as the most relevant dysregulated category, with inhibition of viability and survival-associated functions and upregulation of cell death connected functions (***Table S6***). The two functions with the highest |Z-scores| were *Cell Viability of Tumor Cell Lines* (Z-score = -3.503) and *Apoptosis* (Z-score = 2.987) (p-values < 0.001), and this was the result of the significant dysregulation of 134 and 341 molecules, respectively (***Table S6***). With the *Regulator Effects* IPA function, a network leading to a predicted strong inhibition of *Cell Viability of Tumor Cell Lines* and a strong activation of *Apoptosis* and *Cell Death of Tumor Cell lines* (p-values < 0.001)

(***Fig. S7***) was identified, therefore linking the most relevant functions already detected. This analysis enabled the selection of potentially especially relevant molecules that were significantly downregulated and impacted all three functions: *Ccnb1, Nuf2, Plat, Dhcr24, Rrm2, Pbk, Foxm1, Ezh2, Pim1, Snai2, Spp1* and *Aurka*. Two molecules significantly downregulated in this cell line acted as upstream regulators in this network: *Vgll3* (which led to the downregulation of *Spp1* and *Snai2*) and *Myb* (which impacted all three functions depicted and directly regulated many of the other molecules in the network) (***Fig. S7***).

In the *Canonical Pathways* analysis with the B16F10 miR-320a-3p$^+$ SB cell line, *NER* was the most dysregulated pathway considering p-values and Z-scores (as seen with B16F10 miR-666-3p$^+$ SB cells), showing the highest |Z-score| (-2.121), followed by *Aryl Hydrocarbon Receptor Signaling* which had a much lower absolute value (Z-score = -1) (both p-values < 0.001). In the *NER Pathway*, the different steps in the repair mechanism were predicted to be downregulated and, as seen with B16F10 miR-666-3p$^+$ SB cells, numerous functions were impacted by this pathway. While *Cell Survival* and *Cell Viability* were predicted to be inhibited, *Apoptosis* and *Cell Death of Tumor cell Lines* were predicted to be activated (p-values < 0.001, ***Fig. S8***), which is correlated to the increased tumor cell death observed in the functional experiments. As in the other cell lines, *Cell Death and Survival* was the most relevant high order category in the *Diseases and Biological Functions* analysis, and within it, the most significantly regulated pathway was *Cell Viability of Tumor Cell Lines*. However, this function was not predicted to be so strongly downregulated in comparison to the other cell lines (Z-score = -1.314, p-value = $1.2x10^{-04}$) and the number of molecules significantly dysregulated within it was also lower (49) (***Table S7***). Finally, the *Regulator Effects* IPA function led to the selection of a network that includes a strong downregulation of the *Excision Repair* pathway, which stresses the relevance of this function already highlighted in the *Canonical Pathway* analysis (***Fig. S9***). IPA tools were used to add various functions that were affected by the network, including *DNA Damage Response of Tumor Cell Lines* and *Cell Viability* (predicted to be inhibited) and *Cell Death of Melanoma Cell Lines/Tumor Cell Lines* (predicted to be activated) (all p-values < 0.001). The only molecule affecting all depicted functions was *Rpa1*, which was downregulated and highlighted as a key miRNA target candidate (***Fig. S9***).

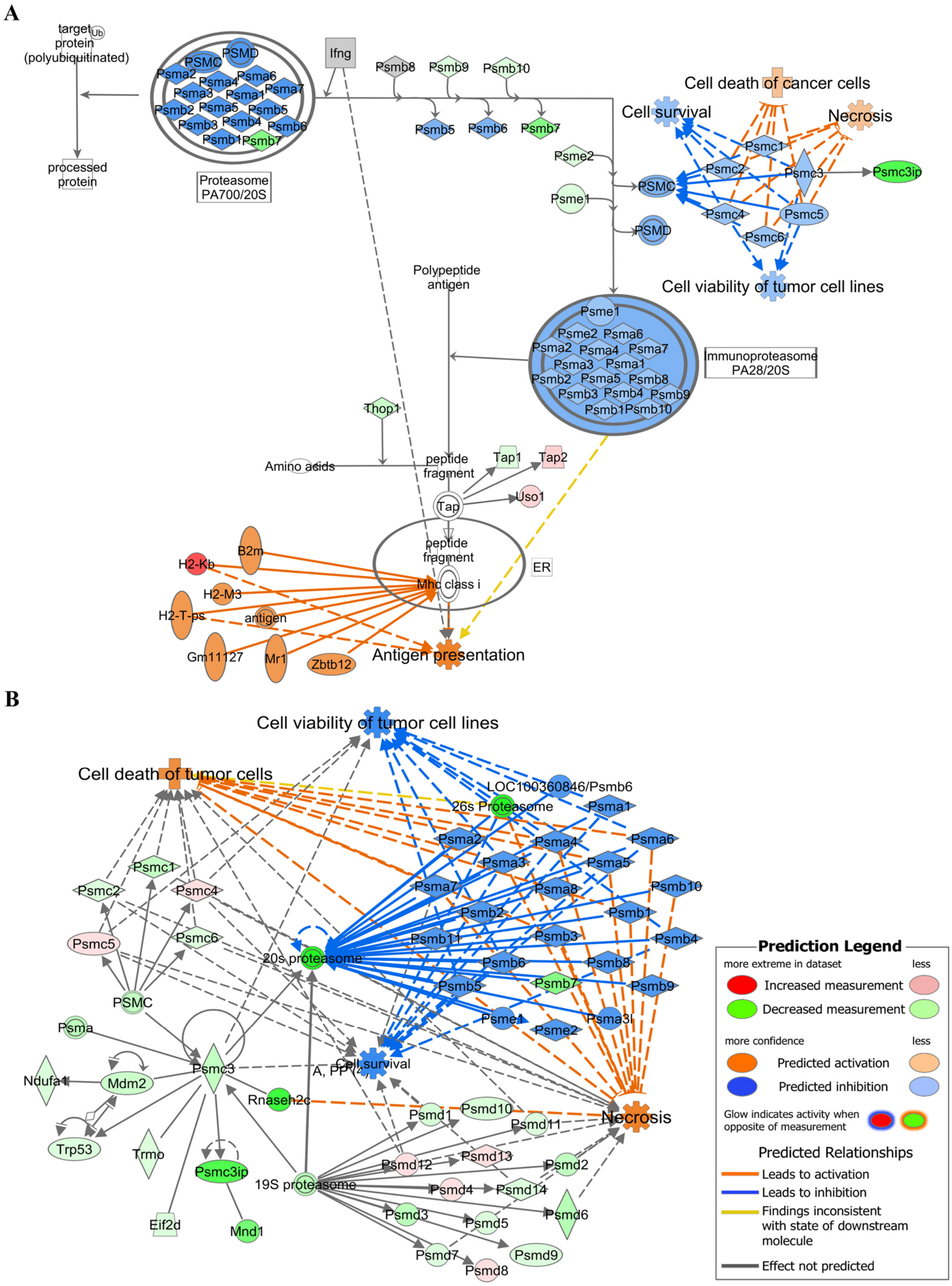

Figure S4: ***Relevant Molecules Analysis* for B16F10 miR-320a-3p^{+} SB. A)** The proteasome components and their connection with antigen presentation are depicted. **B)** A network including the most relevant molecules and complexes that interact with *Psmc3* is shown. Figure based on IPA (Ingenuity Pathway Analysis) graphs, Qiagen. SB: sorted-bulk, *Psmc3:* 26S proteasome regulatory subunit 6A.

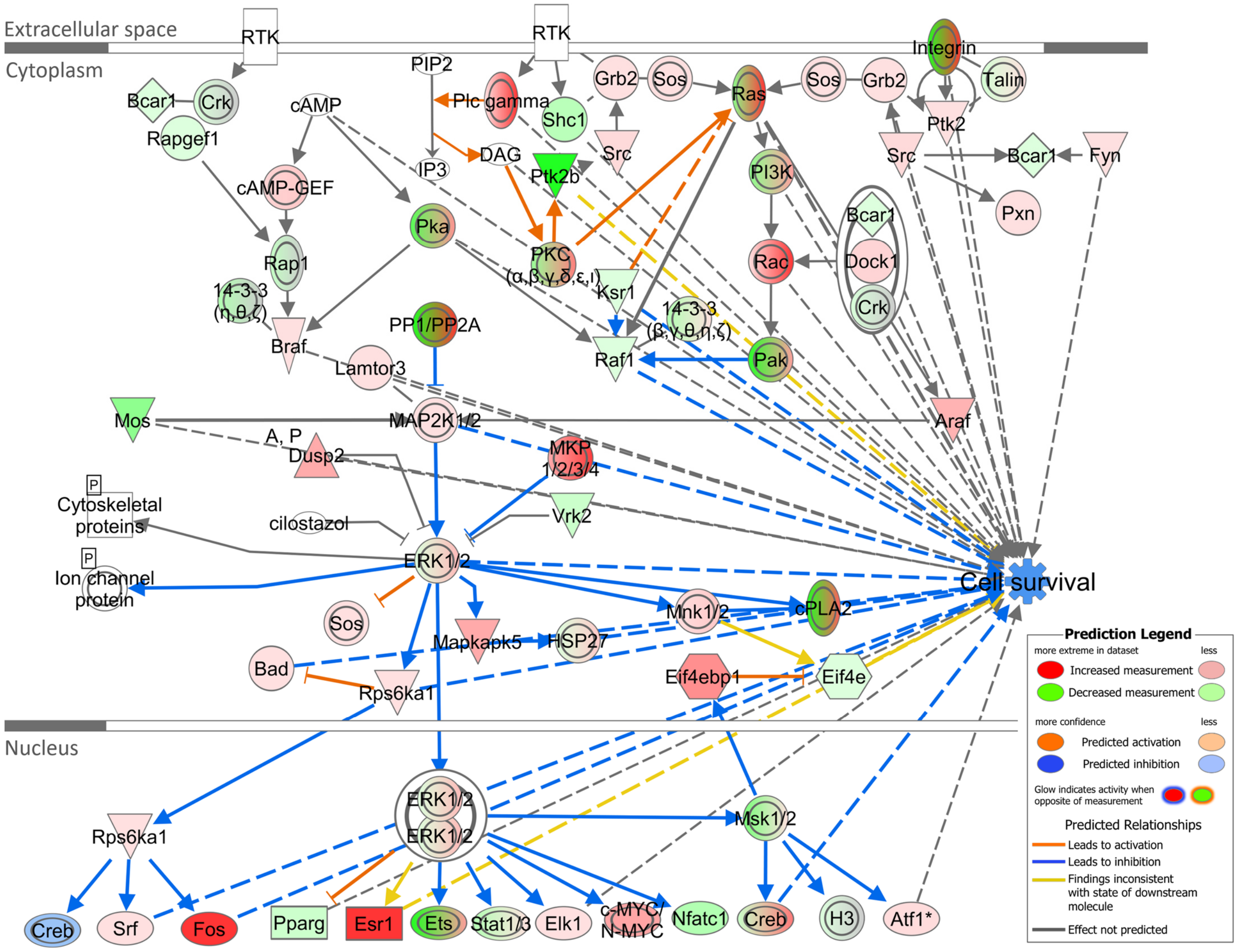

Figure S5: Dysregulated *ERK/MAPK Signaling Pathway* in B16F10 miR-7037-5p+ SB. Figure based on IPA (Ingenuity Pathway Analysis) graphs, Qiagen. ERK/MAPK: mitogen activated protein kinases, SB: sorted-bulk.

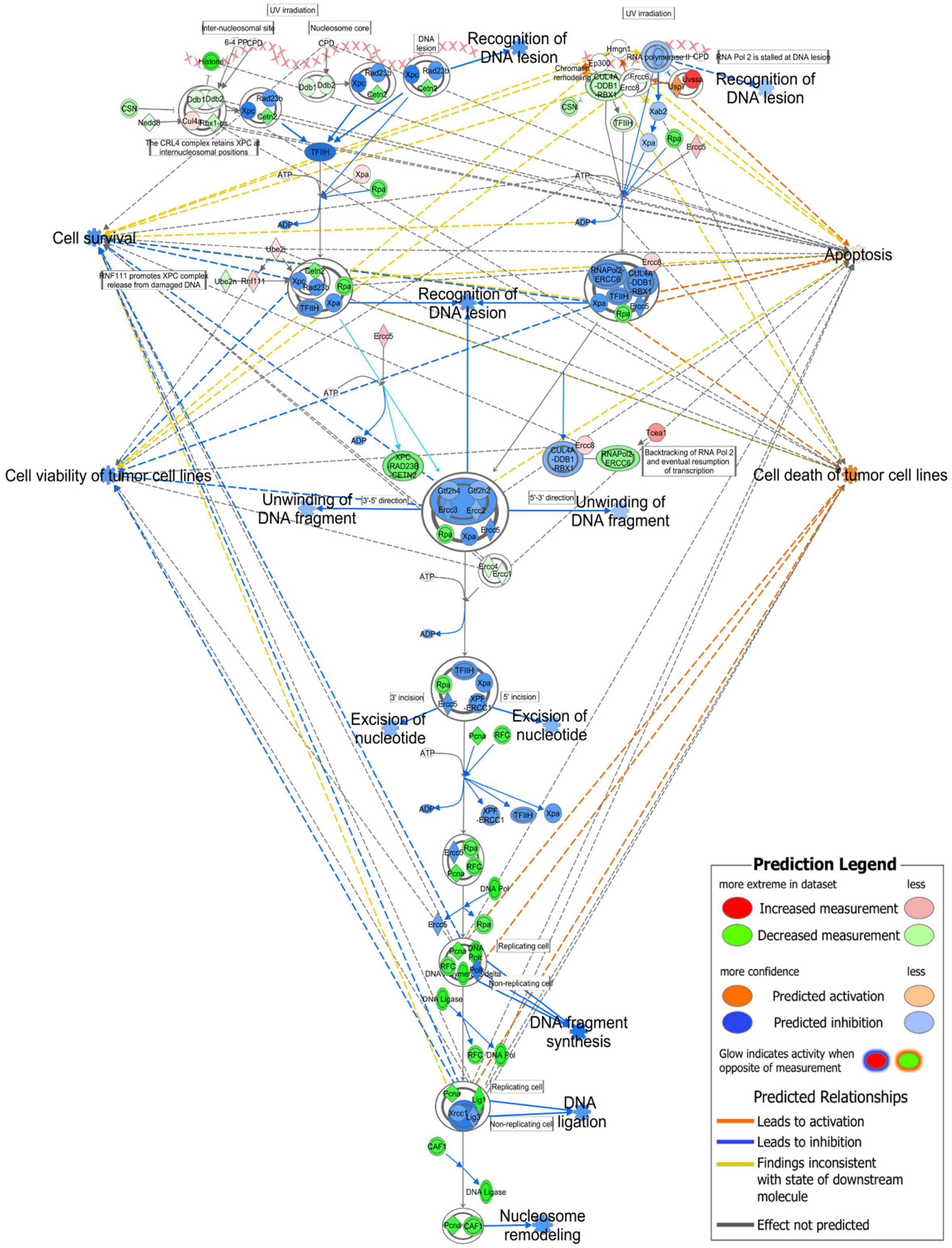

Figure S6: Dysregulated *NER Pathway* in B16F10 miR-666-3p+ SB. Figure based on IPA (Ingenuity Pathway Analysis) graphs, Qiagen. NER: nucleotide excision repair, SB: sorted-bulk.

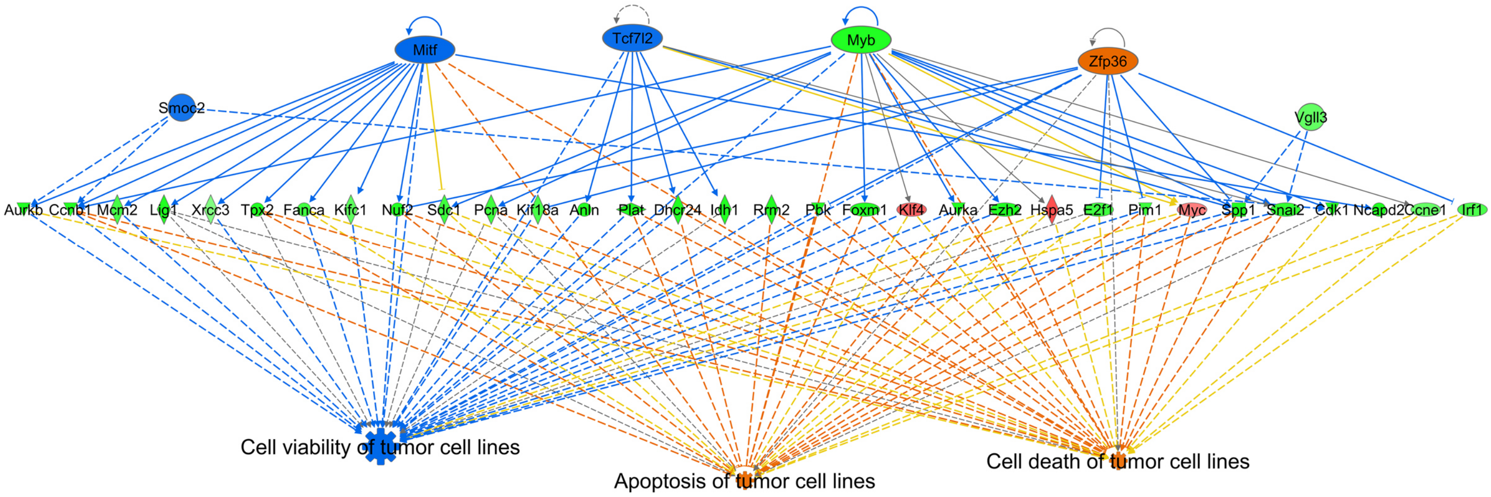

Figure S7: IPA *Regulator Effects* network in B16F10 miR-666-3p+ SB. The most relevant network obtained with this function is shown (p-values for shown annotations < 0.001). Figure based on IPA (Ingenuity Pathway Analysis) graphs, Qiagen. SB: sorted-bulk.

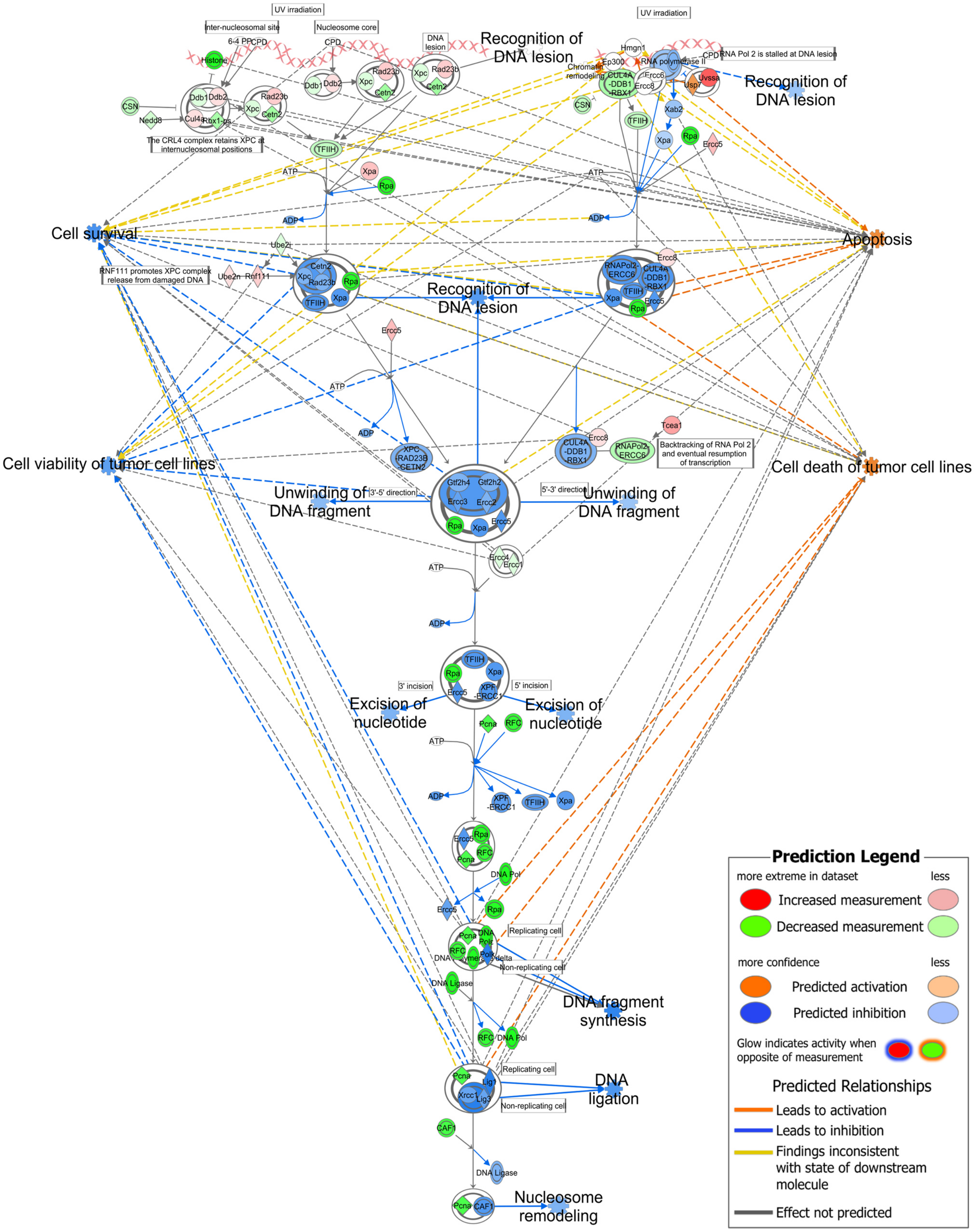

Figure S8: Dysregulated *NER Pathway* in B16F10 miR-320a-3p^{+} SB.

Figure based on IPA (Ingenuity Pathway Analysis) graphs, Qiagen. NER: nucleotide excision repair, SB: sorted-bulk.

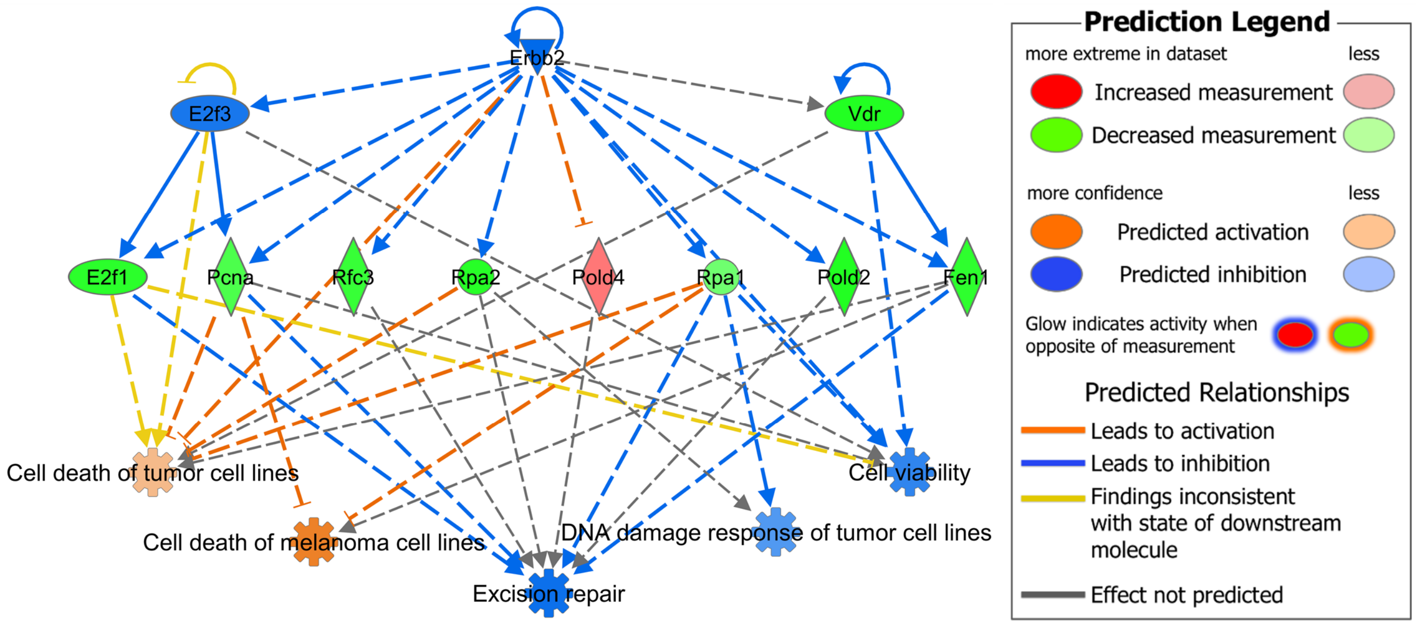

Figure S9: IPA *Regulator Effects* network in B16F10 miR-320a-3p+ SB. The most relevant network obtained with this function is shown (p-values for shown annotations < 0.001). Figure based on IPA (Ingenuity Pathway Analysis) graphs, Qiagen. SB: sorted-bulk.

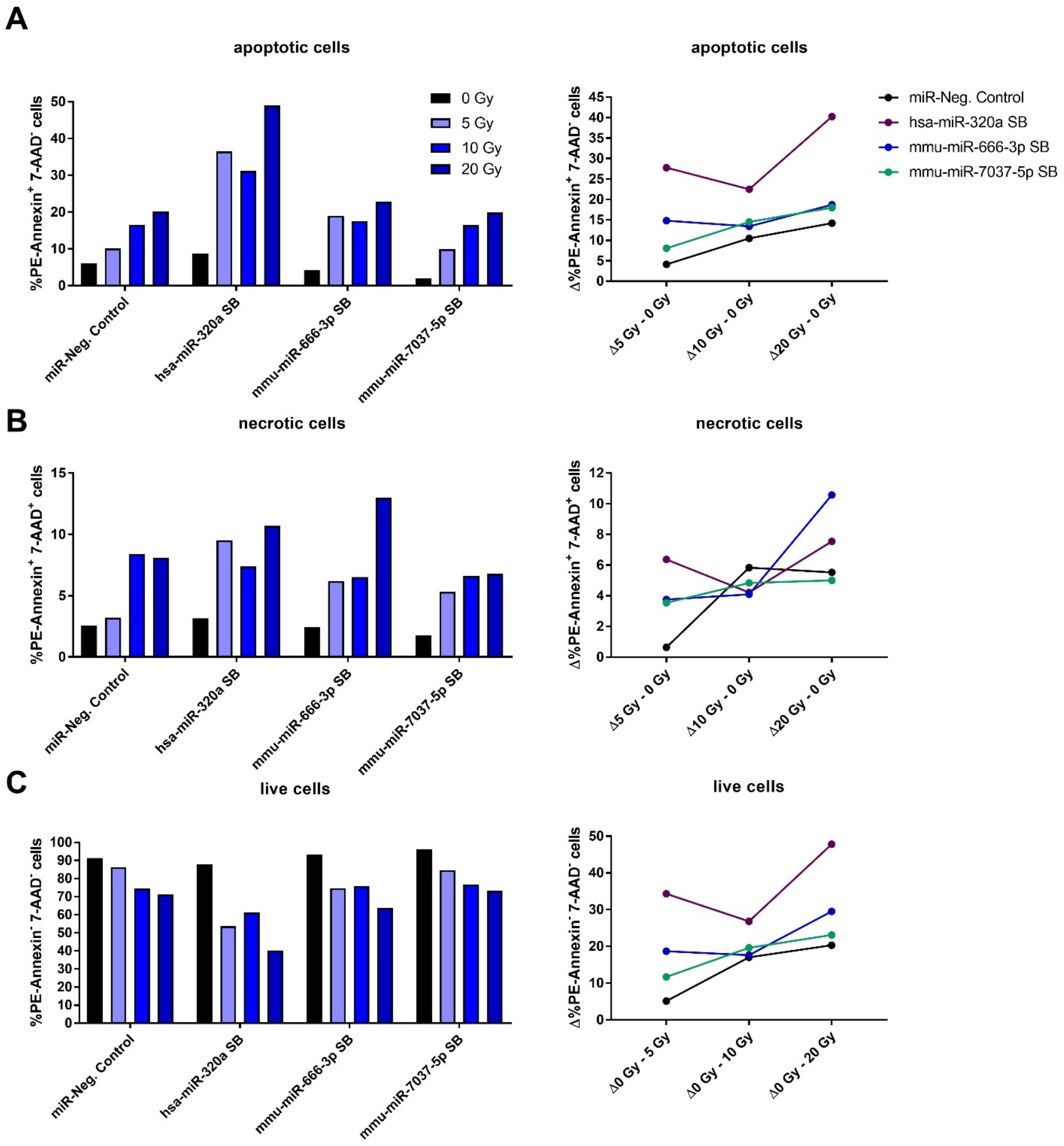

Figure S10: B16F10 miR-320a-3p+ SB and B16F10 miR-666-3p+ SB cell lines show enhanced vulnerability to γ-irradiation. B16F10 miR-Neg. Control, B16F10 miR-7037-5p+ SB, B16F10 miR-320a-3p+ SB and B16F10 miR-666-3p+ SB cell lines were treated with graded γ-irradiation doses and the proportion of apoptotic **(A)** and necrotic **(B)** cells, as well as the frequency of viable cells **(C)** were determined by flow cytometry. SB: sorted bulk.

Tables

Table S1: Preselected miRNA candidates from the screen.

Increased killing miRNA	Z-score with CTLs	Ranking
hsa-miR-34c-5p	5.9	1
hsa-miR-142-5p	5.2	2
hsa-miR-20b-5p	3.0	3
hsa-miR-532-5p	2.7	4
hsa-miR-183-3p	2.7	5
mmu-miR-876-5p	2.3	6
mmu-miR-691	2.2	7
hsa-miR-551b-3p	1.9	8
hsa-miR-301a-3p	1.9	9
mmu-miR-686	1.8	10
hsa-let-7b-5p	1.7	11
mmu-miR-7082-5p	1.5	12
hsa-miR-320a-3p	1.5	13
mmu-miR-669k-5p	1.5	14
mmu-miR-709	1.4	15
mmu-miR-669h-5p	1.4	16
mmu-miR-28b	1.4	17
mmu-miR-666-3p	1.3	18
mmu-miR-5622-5p	1.3	19
mmu-miR-7004-5p	1.3	20
mmu-miR-742-5p	1.3	21
mmu-miR-326-3p	1.3	22
mmu-miR-132-5p	1.3	23
hsa-miR-200c-3p	1.3	24
mmu-miR-6923-3p	1.2	25
hsa-miR-141-3p	1.2	26
mmu-miR-7066-3p	1.2	27
mmu-miR-3078-3p	1.2	28
hsa-miR-370-3p	1.2	29
mmu-miR-5617-3p	1.2	30
hsa-miR-147b	1.1	31
mmu-miR-6363	1.1	32
mmu-miR-5709-3p	1.1	33
mmu-miR-1930-5p	1.1	34
mmu-miR-6916-3p	1.1	35
mmu-miR-7044-5p	1.1	36
mmu-miR-6980-5p	1.1	37
mmu-miR-7037-5p	1.0	38

mmu-miR-211-3p	1.0	39
hsa-let-7d-5p	1.0	40
mmu-miR-688	1.0	41
hsa-miR-339-5p	1.0	42

Decreased killing miRNA	Z-score with CTLs	Ranking
hsa-miR-302a-3p	-1.0	11
mmu-miR-3107-3p	-1.0	10
hsa-miR-27b-3p	-1.1	9
mmu-miR-6941-5p	-1.1	8
hsa-miR-323a-3p	-1.1	7
mmu-miR-5131	-1.2	6
mmu-miR-432	-1.2	5
mmu-miR-1934-5p	-1.3	4
hsa-miR-410-5p	-1.3	3
mmu-miR-5098	-1.5	2
mmu-miR-1897-5p	-2.2	1

CTLs: cytotoxic T lymphocytes.

Note: Human miRNAs listed in this table are conserved in mice.

Table S2: Top miRNA hits list from the screen.

miRNA	Sequence
mmu-miR-7082-5p	UACGGGCAGGAGGAGGGGAGG
hsa-miR-320a-3p	AAAAGCUGGGUUGAGAGGGCGA
mmu-miR-666-3p	GGCUGCAGCGUGAUCGCCUGCU
hsa-miR-200c-3p	UAAUACUGCCGGGUAAUGAUGGA
mmu-miR-326-3p	CCUCUGGGCCCUUCCUCCAGU
mmu-miR-7037-5p	AAGGUGGCCACAGGAGAUCAUGGU
hsa-miR-339-5p	UCCCUGUCCUCCAGGAGCUCACG

Note: Human miRNAs listed in this table are conserved in mice.

Table S3: Relative miRNA overexpression in transduced SB cell lines determined by qPCR. The shown FCs vs. parental cell line are the averages of three different experiments.

Cell line	Average FC vs. parental $2^{(-\Delta\Delta Ct)}$
B16F10 miR-7082-5p$^+$ SB	35340.65
B16F10 miR-320a-3p$^+$ SB	1.74
B16F10 miR-666-3p$^+$ SB	Infinite
B16F10 miR-200c-3p$^+$ SB	2.75
B16F10 miR-326-3p$^+$ SB	2.58
B16F10 miR-7037-5p$^+$ SB	14.18
B16F10 miR-Neg. Control SB	Infinite

Note: Variations in the FC values are mainly due to the differences in the basal endogenous expression of the detected miRNAs in the parental cell line. SB: sorted-bulk, FC: fold change.

Table S4: Number of significantly dysregulated genes in RNA-seq. After applying a cut-off of p-value = 0.001 and $log_2(FC) = |1|$, the selected dysregulated genes were counted for the comparisons between each cell line and the control cell line B16F10 miR-Neg. Control (clone M).

Cell line	Total dysregulated genes
B16F10 miR-320a-3p$^+$ SB	578
B16F10 miR-666-3p$^+$ SB	1633
B16F10 miR-7037-5p$^+$ SB	816

FC: fold change, SB: sorted-bulk.

Table S5: Dysregulated *Diseases and Biological Functions* within the category *Cell Death and Survival* in B16F10 miR-7037-5p+ SB. These low order functions are sorted according to decreasing activation Z-scores.

Diseases or functions annotation	p-value	Activation Z-score	N° of Molecules
Apoptosis of Tumor Cell Lines	0.000088	2.185	88
Apoptosis	$8.37x10^{-08}$	2.131	179
Necrosis	$2.05x10^{-05}$	0.859	167
Cell Viability	$2.34x10^{-06}$	-0.906	103
Cell Survival	$2.72x10^{-08}$	-1.13	115
Cell Viability of Tumor Cell Lines	$3.35x10^{-05}$	-1.909	70
Survival of Saccharomyces Cerevisiae	$2.24x10^{-05}$	NP	4

Note: The functions specific for cell types that are not relevant in our system were disregarded. Table adapted from IPA (Ingenuity Pathway Analysis) results, Qiagen. SB: sorted-bulk, NP: no predicted regulation.

Table S6: Dysregulated *Diseases and Biological Functions* within the category *Cell Death and Survival* in B16F10 miR-666-3p+ SB. These low order functions are sorted according to decreasing activation Z-scores.

Diseases or functions annotation	p-value	Activation Z-score	N° of Molecules
Apoptosis	$3.42x10^{-17}$	2.987	341
Necrosis	$6.82x10^{-17}$	2.854	340
Cell Death of Tumor Cell Lines	$1.94x10^{-12}$	2.704	214
Apoptosis of Tumor Cell Lines	$1.95x10^{-08}$	2.678	163
Cell Death of Cervical Cancer Cell Lines	$6.49x10^{-08}$	1.84	58
Cell Death of Fibroblast Cell Lines	$5.81x10^{-07}$	-0.632	61
Cell Death of Connective Tissue Cells	$5.16x10^{-09}$	-0.768	87
Cell Survival	$5.08x10^{-14}$	-2.668	208
Cell Viability	$1.05x10^{-12}$	-2.71	195
Cell Viability of Tumor Cell Lines	$2.28x10^{-10}$	-3.503	134

Note: The functions specific for cell types that are not relevant in our system were disregarded. Table adapted from IPA (Ingenuity Pathway Analysis) results, Qiagen. SB: sorted-bulk.

Table S7: Dysregulated *Diseases and Biological Functions* within the category *Cell Death and Survival* in B16F10 miR-320a-3p+ SB. These low order functions are sorted according to decreasing activation Z-scores.

Diseases or functions annotation	p-value	Activation Z-score	N° of Molecules
Apoptosis of Tumor Cell Lines	2.61×10^{-05}	0.494	65
Cell Death of Tumor Cell Lines	2.79×10^{-07}	0.27	85
Apoptosis	5.14×10^{-10}	0.022	135
Cell Death of Lymphoblastoid Cell Lines	0.000194	-0.092	9
Cell Viability of Epithelial Cell Lines	9.53×10^{-07}	-0.178	13
Necrosis	3.14×10^{-11}	-0.388	139
Apoptosis of Lymphoblastoid Cell Lines	0.000117	-0.625	6
Cell Death of RPE Cells	0.000196	-0.655	4
Cell Death of Connective Tissue Cells	0.000158	-0.956	33
Cell Survival	9.68×10^{-08}	-1.015	82
Cell Death of Breast Cancer Cell Lines	5.94×10^{-05}	-1.072	24
Cell Viability	7.59×10^{-07}	-1.078	76
Cell Viability of Tumor Cell Lines	0.000119	-1.314	49
Apoptosis of Connective Tissue Cells	0.000241	-1.384	19
Loss of Exocrine Cells	1.51×10^{-05}	NP	4

Note: The functions specific for cell types that are not relevant in our system were disregarded. Table adapted from IPA (Ingenuity Pathway Analysis) results, Qiagen. SB: sorted-bulk, NP: no predicted regulation.

Table S8: *Regulator Effects* highlighted and shared molecules analysis. The molecules from each of the IPA *Regulator Effects* networks analyzed are shown. The references indicate especially relevant molecules and the ones that participated in more than one *Regulator Effects* network.

Cell line	All molecules in the analyzed *Regulator Effects* network
**B16F10 miR-7037-5p+ SB**	***Aurka***, *Col4a1*, ***Fn1***, ***Pbk***, ***Spp1***, *Tyms*, *Vim*, *S100a6*, *Sav1*.
**B16F10 miR-666-3p+ SB**	*Anln*, ***Aurka***, *Aurkb*, ***Ccnb1***, *Ccne1*, *Cdk1*, ***Dhcr24***, *E2f1*, ***Ezh2***, *Fanca*, ***Foxm1***, *Hspa5*, *Idh1*, *Irf1*, *Kif18a*, *Kifc1*, *Klf4*, *Lig1*, *Mcm2*, *Myc*, ***Nuf2***, ***Pbk***, *Pcna*, ***Pim1***, ***Plat***, ***Rrm2***, *Sdc1*, ***Snai2***, ***Spp1***, *Tpx2*, *Xrcc3*, *Ncapd2*, *Mitf*, ***Myb***, *Smoc2*, *Tcf7l2*, ***Vgll3***, *Zfp36*.
**B16F10 miR-320a-3p+ SB**	*E2f1*, *Fen1*, *Pcna*, *Pold2*, *Pold4*, *Rfc3*, ***Rpa1***, *Rpa2*, *E2f3*, *Erbb2*, *Vdr*.

**References**
**Bold:** Highlighted molecule
Red: Molecule shared by different cell lines

SB: sorted-bulk, IPA: Ingenuity Pathway Analysis.

Table S9: IPA networks from the pre-selected screen enriched target genes. The networks 1, 2 and 3 are the ones that were relevant for further analysis as they include many of the enriched target genes from the screen.

Network ID	Molecules in network	Score	Enriched target genes	Top diseases and functions
1	A*	20	10	Cellular Development, Hematological System Development and Function, Hypersensitivity Response
2	B*	18	9	Cell Cycle, Gene Expression, Cellular Response to Therapeutics
3	C*	11	6	Carbohydrate Metabolism, Small Molecule Biochemistry, Lipid Metabolism
4	DIO2, TAF7L, TSKS	2	1	Amino Acid Metabolism, Auditory and Vestibular System Development and Function, Cellular Development
5	CST5, E2F4, MRTO4	2	1	Digestive System Development and Function, Organ Morphology, Cell Cycle
6	ADCYAP1, MBTD1, OTX2, SLC6A20	2	1	Nervous System Development and Function, Organ Morphology, Organismal Development

A*: AP4B1, CD19, Cd200r3, Ces1f, CLEC11A, CST7, Cyp2c40 (includes others), EPHB6, ERK1/2, EVI5, Fcrls, GATA2, GPR171, IL4, IL10RA, JPH2, MCTP1, MOS, MYO1E, NFkB (complex), PTN, RAB44, RGS18, RNF112, SERPINB13, SH3GLB2, SLC22A16, SLC8A1, STAB2, TAL1, Tlr11, TMEM14C, TREM3, UBXN1, USP10.
B*: CARS, CCND1, CEBPB, CRYBB2, DDIAS, DNASE1, EIF2AK3, ESRP2, FAM83D, FKBP6, FN1, FTL, GAL3ST1, MDM2, MEG3, mir-500, mir-605, miR-194-5p (miRNAs w/seed GUAACAG), MLF2, NDUFA1, NOP53, PLAC1, PNLIP, PSMC3, PSRC1, PYHIN1, RPS25, SP1, TGFB1, TP53, TUBB6, UBL5, USP42, Zfp871, ZNF668.
C*: arginase, ASL, Ces, CPS1, CROT, Cyp2a12/Cyp2a22, Cyp2c54 (includes others), ECH1, fructose 1,6 biphosphatase, GNG3, GSTA3, GSTP1, GSTT2/GSTT2B, ISL1, Krtap4-1/Krtap4-2, Krtap6-3, LEP, LIPC, MGST3, NFE2L2, NPBWR1, NPFF, NPY, NPY5R, PAQR7, PIP5K1C, PLA2G1B, PPARA, SC5D, SLC27A4, SORD, TALDO1, TEF, UGT1A7 (includes others), UTS2.
Table adapted from IPA (Ingenuity Pathway Analysis) results, Qiagen.

Table S10: Summary of the *in silico* analysis of the selected top ranked enriched target genes. These genes are targeted by six miRNAs that increased the killing in the screen (IK miRNAs) and no miRNAs that had no effect on killing (NE miRNAs).

Target gene	IK miRNAs	NE miRNAs	p-value	Odds ratio	RNA-seq (mean TPM)	RNA-seq (mean T/cell)	HPA, protein expression in cancer patients
Ftl1	6	0	0.0011	Inf.	4196.5	839.3	Expressed in melanoma and other cancer types
Ndufa1	6	0	0.0011	Inf.	308.4	61.7	
Psmc3	6	0	0.0011	Inf.	164.9	33.0	
Tubb6	6	0	0.0011	Inf.	119.5	23.9	

TPM: transcripts per million, HPA: Human Protein Atlas.

Table S11: siRNA knockdown of selected target genes measured with RT-qPCR. These results are representative of three different experiments.

siRNA treatment (100 nM)	FC vs. siRNA Negative Control, $2^{(-\Delta\Delta Ct)}$	Knockdown (%)
siRNA a. *Ftl1*	0.28	71.8
siRNA a. *Ndufa1*	0.27	72.5
siRNA a. *Psmc3*	0.49	50.8
siRNA a. *Tubb6*	0.35	65.5
siRNA AllStars Negative Control	1.00	0.0

FC: fold change.

Table S12: Online resources for sequencing data.

Type	Experiment/patients	Source
B16F10 RNA-seq	ERX386688: Illumina HiSeq 2000 sequencing<br>ERX386689: Illumina HiSeq 2000 sequencing<br>ERX386690: Illumina HiSeq 2000 sequencing	SRA, NCBI, U.S. National Library of Medicine[10]
miRNA-seq and clinical data	Melanoma patients available in the TCGA program in August 2019	NIH, National Cancer Institute[11]

SRA: Sequence Read Archive, TCGA: The Cancer Genome Atlas.

Table S13: Cell culture media.

Medium	Composition
Basic RPMI medium	RPMI 1640 (1x) + GlutaMAX + 10% (v/v) FBS
PS RPMI medium	Basic RPMI medium + 100 Units/mL penicillin + 100 µg/mL streptomycin
Puromycin RMPI medium	PS RPMI medium + puromycin 1 µg/mL
Geneticin RPMI medium	PS RPMI medium + geneticin 0.8 mg/mL
Geneticin + Puromycin RPMI medium	PS RPMI medium + geneticin 1 mg/mL + puromycin 1 µg/mL
Hygromycin + Geneticin + Puromycin RPMI medium	Geneticin + Puromycin RPMI medium + hygromycin 100 µg/mL
Complete T cell medium	Minimum Essential Medium Eagle (alpha modification with sodium bicarbonate) + 10% (v/v) FBS + 100 Units/mL penicillin + 100 µg/mL streptomycin + 2.27% (v/v) of culture supernatant from concavalin A stimulated rat splenocytes + 2.27% (v/v) of 0,5 M methyl α-D-mannopyranoside (αMM) + 2 mM L-glutamine + 50 µM 2-mercaptoethanol

Table S14: RT-qPCR Primers.

Target	Primer	Sequence 5' to 3'	Amplicon length (bp)
Ftl1	Forward	GATGGGCAACCATCTGACCA	91
	Reverse	GAGATACTCGCCCAGAGATCC	
Psmc3	Forward	CAAGTGAAGGTAATTGCAGCCA	257
	Reverse	CTGCGCAATGCGATCATACC	
Ndufa1	Forward	CTCGCCATTATGGGGGTGTG	175
	Reverse	CCAGGCCCTTGGACACATAGT	
Tubb6	Forward	AACTCTTCCGGCCTGACAAC	78
	Reverse	CCTCCGTGTAGTGACCCTTG	
β-actin	Forward	ACCCTAAGGCCAACCGTGA	193
	Reverse	ATGGCGTGAGGGAGAGCATA	

bp: base pairs.

References

1. Lei J, Osen W, Gardyan A, et al. Replication-Competent Foamy Virus Vaccine Vectors as Novel Epitope Scaffolds for Immunotherapy. *PLoS One*. 2015;10(9):e0138458. doi:10.1371/journal.pone.0138458
2. FASTX-Toolkit 0.0.13. http://hannonlab.cshl.edu/fastx_toolkit/index.html.
3. Heinz S, Benner C, Spann N, et al. Simple combinations of lineage-determining transcription factors prime cis-regulatory elements required for macrophage and B cell identities. *Mol Cell*. 2010;38(4):576-589. doi:10.1016/j.molcel.2010.05.004
4. Dobin A, Davis CA, Schlesinger F, et al. STAR: ultrafast universal RNA-seq aligner. *Bioinformatics*. 2013;29(1):15-21. doi:10.1093/bioinformatics/bts635
5. Broad Institute. Picard. https://broadinstitute.github.io/picard/.
6. Liao Y, Smyth GK, Shi W. featureCounts: an efficient general purpose program for assigning sequence reads to genomic features. *Bioinformatics*. 2014;30(7):923-930. doi:10.1093/bioinformatics/btt656
7. Frankish A, Diekhans M, Ferreira A-M, et al. GENCODE reference annotation for the human and mouse genomes. *Nucleic Acids Res*. 2019;47(D1):D766-D773. doi:10.1093/nar/gky955
8. Love MI, Huber W, Anders S. Moderated estimation of fold change and dispersion for RNA-seq data with DESeq2. *Genome Biol*. 2014;15(12):550. doi:10.1186/s13059-014-0550-8
9. Khandelwal N, Breinig M, Speck T, et al. A high-throughput RNAi screen for detection of immune-checkpoint molecules that mediate tumor resistance to cytotoxic T lymphocytes. *EMBO Mol Med*. 2015;7(4):450-463. doi:10.15252/emmm.201404414
10. [dataset]National Center for Biotechnology Information; U.S. National Library of Medicine. B16F10 RNA-seq: ERX386688: Illumina HiSeq 2000 sequencing; ERX386689: Illumina HiSeq 2000 sequencing; ERX386690: Illumina HiSeq 2000 sequencing. Sequence Read Archive (SRA). https://www.ncbi.nlm.nih.gov/sra.
11. [dataset]National Cancer Institute; NIH. miRNA-seq and clinical data from melanoma patients. The Cancer Genome Atlas Program. https://www.cancer.gov/about-nci/organization/ccg/research/structural-genomics/tcga. Published 2019. Accessed August 1, 2019.
